# Natural Polymer Nanocomposites Reinforced with Ceramic Nanofillers for Flexible Energy Storage: A Review

**DOI:** 10.3390/nano16181148

**Published:** 2026-09-14

**Authors:** Susana Devesa

**Affiliations:** CEMMPRE, ARISE, Department of Mechanical Engineering, University of Coimbra, Rua Luís Reis Santos, 3030-788 Coimbra, Portugal; sdevesa@dem.uc.pt

**Keywords:** natural polymers, ceramic nanofillers, flexible energy storage, supercapacitors, batteries, biopolymer nanocomposites, sustainability

## Abstract

The growing demand for flexible and wearable electronics has stimulated the development of sustainable energy-storage materials capable of maintaining electrochemical performance under mechanical deformation. Natural polymers are attractive candidates owing to their renewability, low toxicity, biodegradability, and structural versatility; however, their limited electrical conductivity and electrochemical activity often require functional reinforcement. This review critically examines natural polymer–ceramic nanocomposites developed for flexible batteries and supercapacitors, considering their use as electrodes, electrolytes, and separators. A systematic literature search identified 41 studies in which the natural polymer remained a constituent of the final functional composite, while systems employing natural polymers solely as sacrificial templates were excluded. Cellulose emerges as the most widely explored matrix, combined with a broad range of ceramic materials, including MnO_2_, ZnO, SnO_2_, TiO_2_, Fe_3_O_4_, BaTiO_3_, and other functional oxides and inorganic compounds. Ceramic incorporation generally improves electrochemical activity, ion transport, thermal stability, or mechanical integrity, although the reported performance strongly depends on composition, architecture, and device configuration. A major limitation across the literature is the lack of standardized mechanical and flexibility testing, which hinders meaningful comparison between systems. Future progress will require the development of multifunctional, genuinely sustainable architectures together with standardized electrochemical–mechanical testing, biodegradability and end-of-life assessments, and scalable manufacturing strategies.

## 1. Introduction

Modern electronics have witnessed rapid advances in flexible and wearable devices, such as roll-up displays, wearable sensors, miniature robots, smart clothing, foldable smartphones, and implantable medical devices. These types of devices require more sophisticated power systems than conventional portable equipment, as wearable electronics should operate efficiently and safely under different bending, twisting, and stretching conditions. Consequently, conventional rigid energy storage solutions are not suitable for these applications. This gap has accelerated innovation in flexible energy storage devices (FESDs) to meet the energy storage requirements of various flexible products, with research focusing not only on achieving high energy and power densities, long lifetimes, excellent mechanical flexibility, and safety, but also on developing lightweight, cost-effective, and more sustainable solutions [1,2,3].

Among the various energy-storage technologies, batteries and supercapacitors are the two main types of electrochemical energy-storage devices. Batteries have been in the spotlight in academia and industry due to their high energy densities, while supercapacitors are well suited for applications requiring high power density [3].

The principles of flexible batteries and supercapacitors are the same as their conventional rigid counterparts [2], with both relying on electrochemical processes, although distinct electrochemical mechanisms determine their respective energy and power densities. These electrochemical processes give rise to their different charge-storage properties [4]. Batteries store electrical energy through Faradaic electrochemical reactions occurring at the electrode–electrolyte interface. During charge and discharge, ions migrate through the electrolyte and are inserted into (or extracted from) the bulk structure of the electrode materials, while electrons flow through the external circuit. Because charge storage involves diffusion-controlled redox reactions within the bulk of the electrode materials, batteries generally exhibit high energy densities but relatively lower power densities than supercapacitors [5]. Electric double-layer capacitors (EDLCs), one of the main types of supercapacitors, store charge through the adsorption of electrolyte ions onto the surface of electrode materials. No redox reactions are required, so the response to changes in potential without diffusion limitations is rapid and leads to high power. However, because the charge is confined to the surface, the energy density of supercapacitors is lower than that of batteries [4].

**Figure 1:**
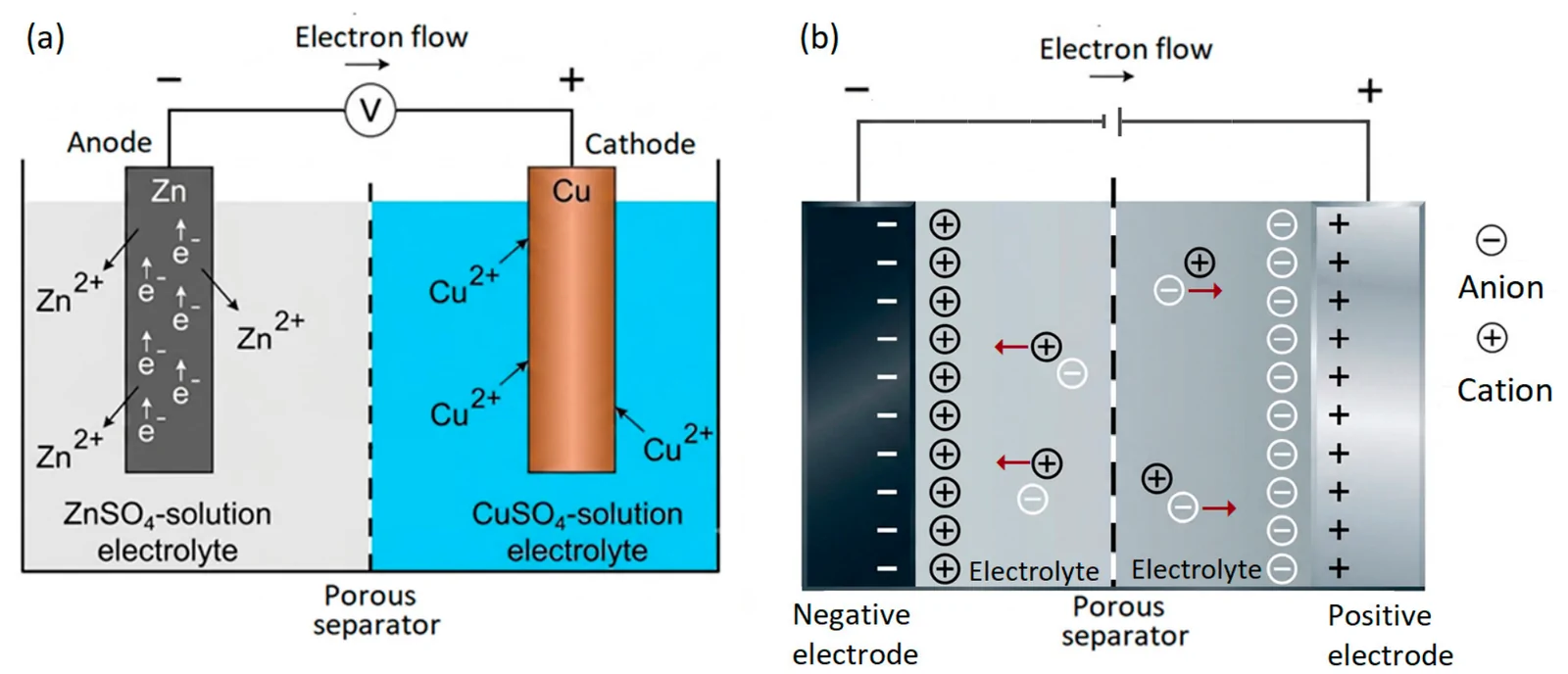
Schematic comparison of the charge-storage mechanisms of (**a**) a Daniell cell (battery) and (**b**) an electric double-layer capacitor (EDLC). Adapted from [5,7].

Figure 1 compares the charge-storage mechanisms of batteries and EDLCs, showing that both devices consist of two electrodes in contact with an electrolyte. Figure 1a presents a schematic representation of a Daniell cell, illustrating the fundamental operating principles of a battery. The cell consists of two half-cells separated by an ion-permeable membrane (or a salt bridge), which allows ionic conduction while preventing the direct mixing of the electrolytes. One half-cell contains a zinc electrode immersed in a zinc sulfate solution, whereas the other contains a copper electrode immersed in a copper sulfate solution. During discharge, the zinc anode undergoes oxidation (Zn → Zn^2+^ + 2e^−^), releasing electrons that flow through the external circuit to the copper cathode, thereby generating an electric current. Simultaneously, copper ions in the cathode compartment are reduced (Cu^2+^ + 2e^−^ → Cu) and deposited onto the copper electrode. To maintain electrical neutrality, ions migrate through the membrane or salt bridge. The cell continues to deliver electrical energy until one of the electroactive species is depleted or the electrochemical reaction reaches equilibrium [6].

EDLCs, as shown in Figure 1b, generally consist of two porous electrodes, typically based on activated carbon, separated by an ion-permeable separator and immersed in an electrolyte. Energy is stored electrostatically through a non-faradaic mechanism, in which no electron transfer or redox reactions occur across the electrode/electrolyte interface. Instead, charge is stored through the electrostatic adsorption of electrolyte ions at the electrode surface [5,7,8].

In contrast, pseudocapacitors, another class of supercapacitors, store energy through fast and reversible faradaic redox reactions occurring at or near the electrode surface. Because these charge-transfer reactions are considerably faster than the diffusion-controlled electrochemical reactions that occur in batteries, pseudocapacitors exhibit higher power density than batteries, although generally lower than that of EDLCs. In EDLCs, energy storage is based on the electrostatic adsorption/desorption of electrolyte ions, a purely physical and highly reversible process that enables exceptionally high power density and excellent cycling stability. By contrast, batteries rely on slower bulk electrochemical reactions and undergo structural and chemical changes in the electrode materials during cycling, resulting in lower power density and shorter cycle life [7].

Although the charge-storage mechanisms of batteries and supercapacitors remain unchanged in their flexible counterparts, the development of flexible energy-storage devices requires significant modifications in materials selection, electrode architecture, and device design to maintain electrochemical performance under repeated mechanical deformation.

To meet the demanding mechanical and electrochemical requirements of flexible energy-storage devices, considerable research has focused on the development of flexible materials for electrodes, electrolytes, separators, current collectors, and packaging materials [3]. Among these, polymeric materials have attracted particular attention owing to their low density, excellent flexibility, ease of processing, and tunable physicochemical properties [9]. Conventional synthetic polymers, such as poly(vinyl alcohol) (PVA), poly(vinylidene fluoride) (PVDF), polyethylene oxide (PEO), and polydimethylsiloxane (PDMS), have been widely employed in flexible energy-storage devices [10].

These synthetic polymers have proven to be suitable materials for meeting the rapidly growing demand for flexible and wearable energy-storage systems. However, their widespread use raises significant environmental concerns. Conventional synthetic polymers exhibit high resistance to aging and biodegradation, leading to their persistence in the environment and contributing to pollution after disposal. Furthermore, most monomers used in the production of plastics, such as ethylene and propylene, are derived from fossil-based feedstocks, thereby contributing to the depletion of non-renewable resources and increasing greenhouse gas emissions. In addition, polymer synthesis often involves hazardous chemicals and may generate toxic by-products. These environmental challenges have intensified the search for sustainable alternatives, driving increasing interest in polymeric materials derived from renewable biological resources [2,11,12].

Among the various bio-based materials under investigation, biopolymers have emerged as promising candidates owing to their renewability, biodegradability, and reduced environmental footprint compared with conventional petroleum-based polymers. Despite the well-established infrastructure and processing technologies available for fossil-based plastics, biopolymers offer a viable pathway towards the development of sustainable flexible energy-storage materials [12,13].

Biopolymers are naturally derived or biologically produced macromolecules composed of repeating structural units. Naturally occurring biopolymers used in flexible energy-storage devices are primarily polysaccharides and proteins. Representative examples include cellulose, chitosan, alginate, starch, silk fibroin, collagen, gelatin, pectin, and hyaluronic acid. Their increasing adoption is driven not only by their renewable origin and low toxicity but also by growing environmental concerns associated with petroleum-derived polymers [12].

In recent years, biopolymers have attracted considerable attention for flexible electrochemical energy-storage applications, serving as electrolytes, separators, binders, electrode matrices, and ion-exchange membranes in batteries and supercapacitors. Their excellent biocompatibility, mechanical flexibility, environmental sustainability, and biodegradability make them particularly attractive for next-generation wearable and transient electronic devices [12].

Despite their distinct origins and environmental impacts, both conventional synthetic polymers and biopolymers share intrinsic limitations, particularly their low electrical conductivity and limited electrochemical activity, which often restrict their direct use as functional materials in energy-storage devices. Consequently, polymer composite materials have become one of the most important material platforms for flexible energy-storage devices. By incorporating conductive or electrochemically active fillers, including carbon nanomaterials, metals, metal oxides, semiconductors, conducting polymers, or ionic conductors, into polymer matrices, these composites combine the mechanical compliance of polymers with enhanced electrical and electrochemical performance, enabling devices capable of maintaining functionality under repeated bending, stretching, or folding [14].

As discussed above, natural polymers have emerged as promising sustainable alternatives for flexible and wearable energy-storage devices due to their renewability, biodegradability, low cost, and attractive physicochemical properties. However, the rapidly expanding literature is dispersed across different natural polymer matrices, ceramic nanofillers, and device configurations, making it difficult to establish clear structure–property–performance relationships. Therefore, a comprehensive overview integrating the different roles of natural polymer–ceramic nanocomposites in flexible batteries and supercapacitors, together with the current challenges and future research opportunities, is timely.

This review provides a comprehensive overview of natural polymer nanocomposites reinforced with ceramic nanofillers for flexible and wearable energy-storage applications. Following a brief discussion of the literature search methodology, the review critically examines recent advances in natural polymer–ceramic nanocomposites. For each class of natural polymer, the fundamental properties relevant to flexible energy-storage devices are first outlined, followed by a discussion of the ceramic nanofillers employed and their effects on the mechanical, electrical, electrochemical, and, where relevant, thermal performance of the resulting nanocomposites. Finally, the review highlights the current challenges and future research perspectives toward the development of high-performance, sustainable, and environmentally friendly materials for next-generation flexible energy-storage devices.

## 2. Literature Search Methodology

### 2.1. Search Strategy

A comprehensive literature search was conducted using the Scopus database to identify publications related to natural polymer nanocomposites reinforced with ceramic nanofillers for flexible and wearable energy-storage applications. The search was performed in July 2026 using combinations of keywords representing natural polymers (cellulose, chitosan, alginate, starch, gelatin, and silk fibroin), ceramic nanofillers (ceramic, metal oxide, nanoparticle, nanofiller, nanoceramic, TiO_2_, titanium dioxide, ZnO, zinc oxide, SiO_2_, silicon dioxide, Al_2_O_3_, aluminum oxide, alumina, BaTiO_3_, barium titanate, hydroxyapatite, HAp, ZrO_2_, zirconia, CeO_2_, ceria, SnO_2_, tin oxide, MgO, magnesium oxide, Fe_3_O_4_, and magnetite), and application-related terms associated with flexible or wearable batteries and supercapacitors. Boolean operators (AND/OR) and wildcard characters (*) were used to retrieve relevant variations of the selected keywords.

The primary search strategy was performed using the TITLE-ABS-KEY field with the following search query:

TITLE-ABS-KEY((cellulose OR chitosan OR alginate OR starch OR gelatin OR “silk fibroin”) AND (ceramic* OR “metal oxide*” OR nanoparticle* OR nanofiller* OR nanoceramic* OR TiO_2_ OR “titanium dioxide” OR ZnO OR “zinc oxide” OR SiO_2_ OR “silicon dioxide” OR Al_2_O_3_ OR “aluminum oxide” OR alumina OR BaTiO_3_ OR “barium titanate” OR hydroxyapatite OR HAp OR ZrO_2_ OR zirconia OR CeO_2_ OR ceria OR SnO_2_ OR “tin oxide” OR MgO OR “magnesium oxide” OR Fe_3_O_4_ OR magnetite) AND (flexible OR wearable) AND (battery* OR supercapacitor*)) AND DOCTYPE(ar).

Only peer-reviewed research articles published in English were considered. Review articles, conference papers, book chapters, editorials, notes, and other non-original publications were excluded from the primary search. Following the electronic search, the titles and abstracts of the retrieved records were screened by the author to assess their relevance to the scope of this review. Studies focusing on natural polymer-based nanocomposites reinforced with ceramic nanofillers for flexible or wearable batteries and supercapacitors were subsequently selected for full-text evaluation.

To ensure comprehensive coverage of the available literature, the reference lists of the selected articles were manually examined to identify additional relevant studies that were not retrieved through the electronic search. The selected publications were then analysed qualitatively, with particular emphasis on the type of natural polymer, ceramic nanofiller, fabrication strategy, application in flexible energy-storage devices, and the resulting mechanical and electrochemical performance.

**Figure 2:**
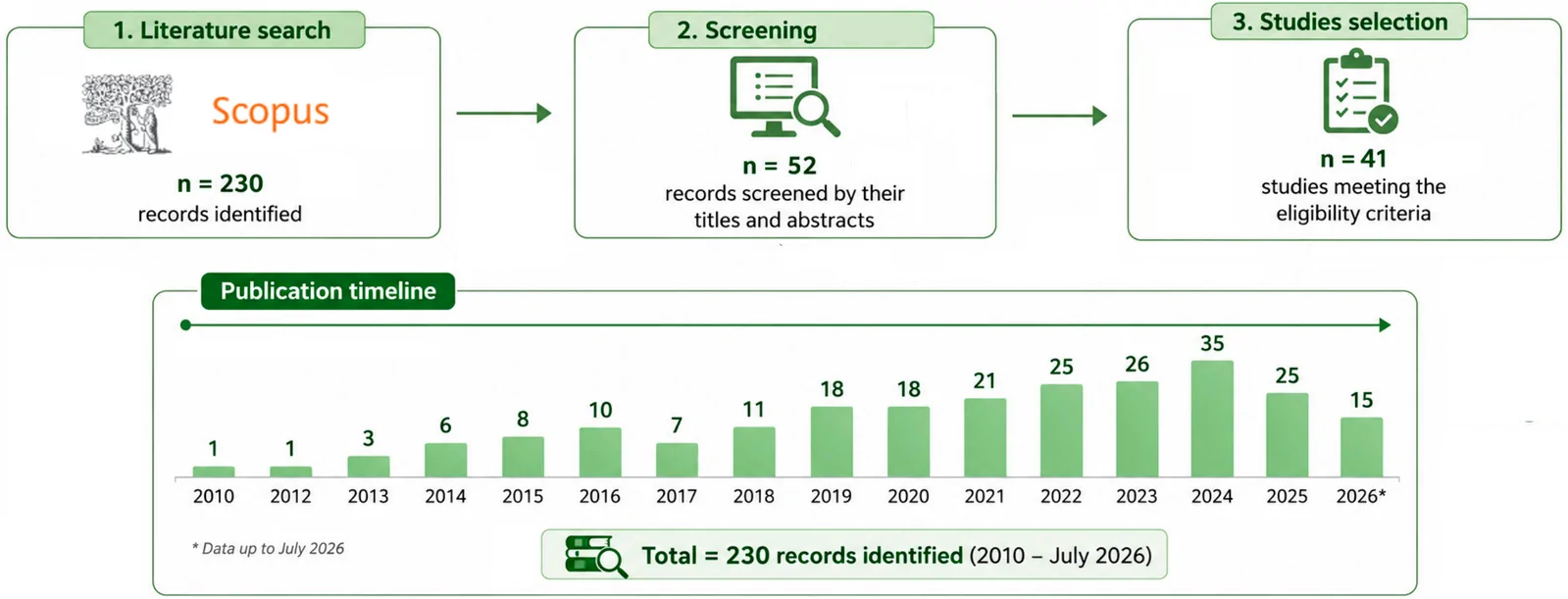
Literature search and study selection workflow adopted in this review, together with the annual distribution of publications included in the Scopus search (2010–July 2026).

An overview of the literature search strategy, study selection workflow, and annual publication trend is presented in Figure 2.

### 2.2. Study Selection

The electronic search retrieved 230 records from the Scopus database (Figure 2). Studies were considered potentially eligible when they investigated natural polymer-based materials reinforced with ceramic nanofillers for flexible or wearable electrochemical energy-storage applications, including batteries and supercapacitors. Articles were excluded if they: (i) focused exclusively on synthetic polymer matrices without relevance to natural polymers; (ii) investigated only non-ceramic fillers, unless included for comparative discussion; (iii) addressed applications unrelated to flexible or wearable energy-storage devices; or (iv) did not provide sufficient information regarding the materials, fabrication strategy, or electrochemical performance. Studies employing additional conductive additives (e.g., carbon nanomaterials or conducting polymers) were considered eligible provided that ceramic nanofillers were incorporated into the natural polymer matrix and played a significant role in the structural or electrochemical performance of the resulting nanocomposite.

In parallel, a preliminary bibliometric assessment was conducted on the complete dataset of 230 records retrieved from Scopus. This analysis indicated a marked increase in scientific output over the last decade, with research activity being predominantly concentrated in Asian countries, particularly China. This geographical distribution reflects the growing global interest in sustainable materials for flexible energy-storage technologies and is further discussed in the concluding remarks.

Following title and abstract screening, 52 studies were selected for full-text assessment. The full texts were subsequently evaluated according to the scope of this review.

In addition, the reference lists of the selected articles were manually screened to identify further relevant publications that were not retrieved through the electronic search. The final set of selected studies, 41, constituted the basis of the qualitative analysis presented in this review.

**Figure 3:**
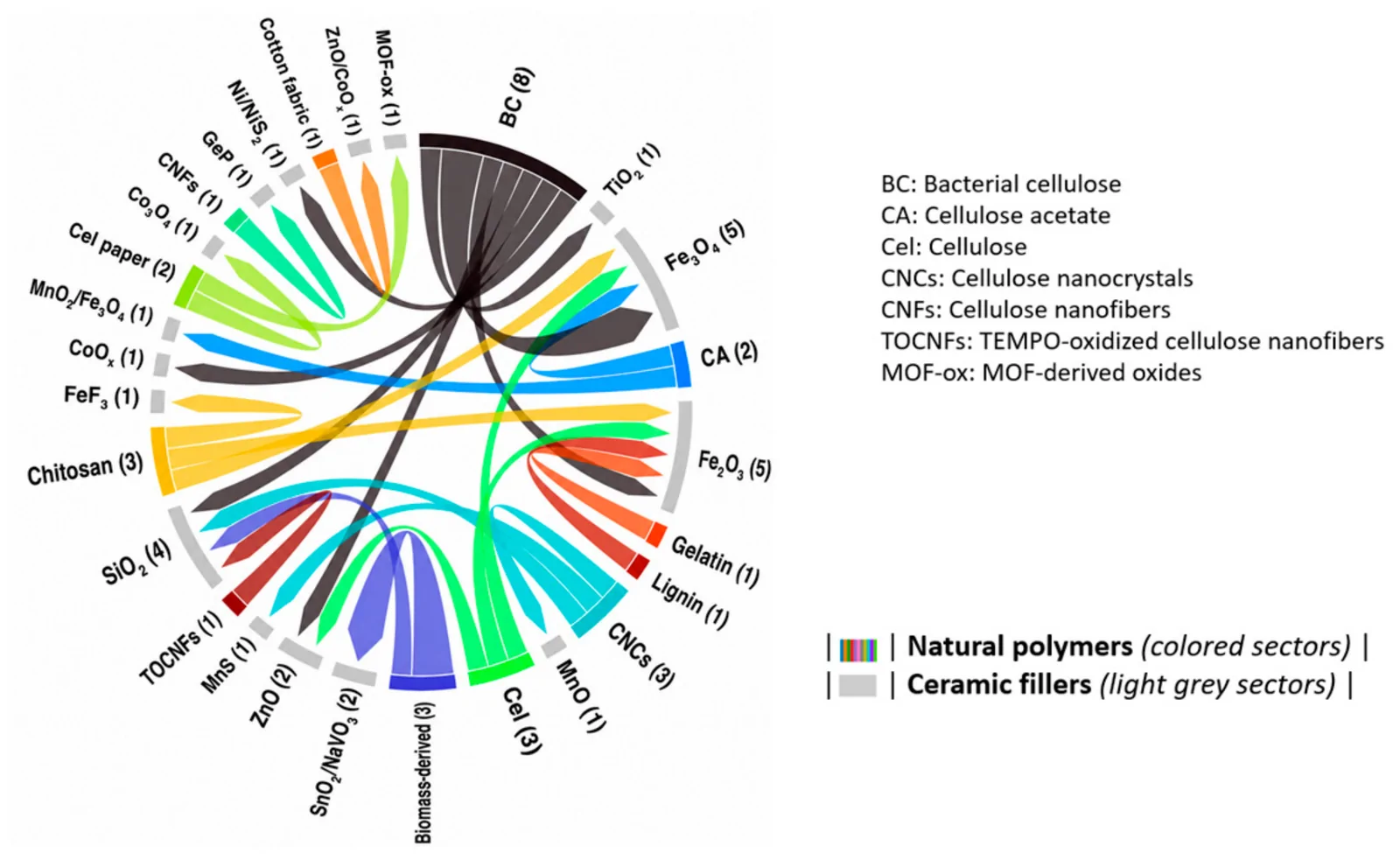
Overview of the natural polymer sacrificial template–ceramic material combinations identified during the title and abstract screening and excluded from the qualitative review because the natural polymer was not retained in the final composite.

Among the studies excluded during the title and abstract screening, a prominent research direction involved the use of natural polymers as sacrificial templates. These studies (n = 29), most of which were based on bacterial cellulose-derived carbon frameworks incorporating Fe_3_O_4_ or Fe_2_O_3_, were excluded because the natural polymer was removed during processing and therefore does not constitute part of the final composite material. Figure 3 summarizes the principal sacrificial natural polymer–ceramic material combinations identified among these excluded studies [15,16,17,18,19,20,21,22,23,24,25,26,27,28,29,30,31,32,33,34,35,36,37,38,39,40,41,42,43].

Table 1 summarizes the combinations of natural polymer matrices, additional polymers, ceramic nanofillers, device components, and target energy storage applications reported in the studies discussed in this review. Specifically, it illustrates the associations between natural polymer matrices (e.g., cellulose, chitosan, alginate, and gelatin) and ceramic nanofillers (e.g., MnO_2_, SnO_2_, TiO_2_, Fe_3_O_4_, and BaTiO_3_), together with the corresponding device component (electrolyte, electrode, or separator) and target application (e.g., lithium-ion batteries, sodium-ion batteries, zinc-air batteries, and supercapacitors).

## 3. Recent Advances in Natural Polymer–Ceramic Nanocomposites for Flexible and Wearable Energy Storage

Among the different components of flexible energy-storage devices, electrodes have received the greatest attention regarding the incorporation of natural polymer–ceramic composites. Natural polymers have been explored as flexible substrates, three-dimensional scaffolds, and structural matrices capable of improving the dispersion of ceramic active materials, enhancing mechanical integrity, and, in some cases, enabling lightweight self-supporting or binder-free electrode architectures. While the vast majority of reported studies concern flexible supercapacitors, the same design principles have also been applied to flexible battery electrodes.

### 3.1. Natural Polymer–Ceramic Nanocomposite Electrodes

#### 3.1.1. Manganese-Based Electrodes

One of the earliest demonstrations of natural polymer–ceramic composite electrodes for flexible energy storage was reported by Jiang et al. [44], who employed commercial rice paper as a renewable cellulose substrate for the fabrication of ternary single-walled carbon nanotubes (SWCNTs)/MnO_2_/cellulose electrodes. Rather than acting solely as a passive support, the cellulose fibres actively participated in the synthesis through their surface hydroxyl groups, which promoted the direct reduction of permanganate ions and the homogeneous deposition of MnO_2_ nanoparticles. The subsequent SWCNTs produced a conductive network interconnecting the MnO_2_ particles, enabling the fabrication of binder-free flexible electrodes with a specific capacitance of 260.2 F g^−1^, an energy density of 9.0 Wh kg^−1^ and only a 12% capacitance loss after 3000 charge–discharge cycles. Moreover, the electrodes exhibited excellent mechanical flexibility, maintaining nearly unchanged capacitance after more than 100 bending cycles, demonstrating that naturally occurring cellulose substrates can simultaneously provide mechanical support, favourable surface chemistry and electrolyte accessibility.

While this work primarily exploited cellulose paper as a chemically active substrate, subsequent research extended the role of cellulose towards the construction of three-dimensional structural frameworks. Yang et al. [45] developed chemically cross-linked cellulose nanocrystal (CNC) aerogels capable of incorporating different electroactive nanoparticles during a one-step sol–gel assembly process, including MnO_2_ (although the authors also demonstrated the incorporation of polypyrrole nanofibers and polypyrrole-coated carbon nanotubes into the same CNC aerogel platform, these systems are beyond the scope of the present review). This bottom-up strategy eliminated the need for binders, carbon/metal supports, and post-synthesis impregnation steps while producing ultralight, highly porous and self-supporting electrodes with active material distributed throughout the CNC network. According to the authors, hydrogen-bonding interactions during assembly facilitated the incorporation of MnO_2_ nanoparticles into the CNC network, while the porous aerogel architecture was proposed to enhance electrolyte diffusion. In addition, the chemically cross-linked CNC network provided remarkable mechanical resilience, maintaining its structural integrity under repeated compression and exhibiting approximately 83% shape recovery after 400 compression cycles in electrolyte. Furthermore, cyclic voltammetry performed during in situ compression revealed only minor changes in capacitive performance. For the MnO_2_-containing aerogels, the voltammograms recorded at 40%, 60%, and 80% compressive strain were essentially indistinguishable, indicating that the electrode maintained its electrochemical response under mechanical deformation. These results illustrate the evolution of cellulose from a flexible substrate to a multifunctional three-dimensional scaffold capable of simultaneously providing structural support, mechanical robustness and efficient ion transport within flexible supercapacitor electrodes.

Despite their different architectures, both studies demonstrate that the role of cellulose extends beyond mechanical support. However, the mechanisms underlying the reported electrochemical performance are not directly comparable. In the rice-paper-based electrode, cellulose provides a chemically active surface that promotes the reduction of permanganate ions and the homogeneous deposition of MnO_2_, while the SWCNTs establish the conductive network. In contrast, in the CNC aerogel, the cellulose nanocrystal network primarily defines the three-dimensional porous architecture, facilitating the incorporation and distribution of MnO_2_ and promoting electrolyte transport. Thus, the improved performance cannot be attributed to a single “cellulose effect”; rather, it emerges from the interplay between polymer surface chemistry, ceramic distribution, pore architecture, and the conductive network. This distinction is important when comparing polymer–ceramic electrodes across different architectures.

The concept of natural polymer-based structural matrices was subsequently extended to hydrogel electrodes by Garcia-Torres et al. [46]. In this work, alginate was combined with poly(3,4-ethylenedioxythiophene) (PEDOT), carbon nanoparticles (CNPs) and MnO_2_ to produce conductive hydrogel electrodes. Hydrogels with different MnO_2_ loadings were prepared by varying the permanganate reduction time from 5 to 60 min, with the highest areal capacitance (42 ± 2 mF cm^−2^) obtained after 30 min, while longer reaction times resulted in a decrease in capacitance because the higher MnO_2_ loading reduced the electrical conductivity of the hydrogel. The interconnected hydrogel network facilitated electrolyte penetration and preserved structural integrity during repeated deformation, whereas PEDOT and CNPs enhanced electrical conductivity by providing effective electron pathways, while MnO_2_ contributed pseudocapacitive behaviour and improved the energy storage capacity, leading to good electrochemical performance. The hydrogel electrode retained approximately 80% of its initial capacitance after 3000 cycles and largely recovered its electrochemical activity after complete folding.

Importantly, the simultaneous presence of alginate, PEDOT, CNPs, and MnO_2_ makes it difficult to isolate the specific contribution of the natural polymer to the electrochemical performance. The decrease in capacitance at higher MnO_2_ loading further demonstrates that increasing the ceramic content does not necessarily improve performance, as excessive MnO_2_ incorporation can reduce the electrical conductivity of the hydrogel. This highlights the importance of optimizing the MnO_2_ loading in relation to the conductive network, rather than treating ceramic content as an independent performance variable.

In addition to energy-storage applications, the multifunctional hydrogel was also demonstrated as a temperature sensor.

Beyond serving as self-supporting hydrogel matrices, natural polymers have also been employed to engineer the microstructure of carbon-based electrodes. Zhang et al. [47] developed flexible chitosan/graphene oxide (GO)/MnO_2_ composite electrodes in which chitosan acted as a structural modifier of the graphene oxide framework while MnO_2_ served as the pseudocapacitive component. The effects of chitosan content and MnO_2_ loading were systematically investigated by varying the chitosan content and the MnO_2_ electrodeposition time, respectively. Chitosan disrupted the restacking of graphene oxide sheets, generating a hierarchical porous network with increased specific surface area, improved wettability and enhanced electrical conductivity. The optimum electrode, prepared using the intermediate chitosan content and the longest MnO_2_ deposition time (20 h), achieved an areal capacitance of 3543.7 mF cm^−2^. According to the authors, the improved electrochemical performance resulted from the synergistic combination of the hierarchical porous carbon framework and the increased MnO_2_ loading, which promoted ion transport, facilitated electron transfer and enhanced pseudocapacitive charge storage. The electrode also exhibited good cycling stability and maintained stable electrochemical performance under repeated bending, demonstrating its suitability for flexible energy-storage applications.

However, the high areal capacitance reported for this architecture (3543.7 mF cm^−2^, †) should be interpreted in the context of its relatively complex electrode composition and the 20 h MnO_2_ electrodeposition used for the optimal electrode. The specific contribution of the chitosan-derived component is therefore difficult to isolate from that of the graphene-derived carbon framework and the deposited MnO_2_. Importantly, this value was obtained in a three-electrode configuration and should therefore not be directly equated with the performance of the complete device. When assembled into an asymmetric solid-state supercapacitor, the system exhibited a substantially lower areal capacitance (1050 mF cm^−2^), together with an energy density of 0.585 mWh cm^−2^ and a power density of 3000 mW cm^−2^. Such differences in composition, measurement configuration, and processing conditions also limit direct quantitative comparison with the cellulose-based systems described above.

An alternative strategy proposed by Zhang et al. [48] further optimized cellulose-based composite electrodes by combining cellulose nanofibres (CNFs), carbon nanotubes (CNTs) and electrochemically deposited MnO_2_ into a flexible self-supporting architecture. The CNF/CNT mass ratio was first optimized to obtain a conductive and mechanically robust substrate, after which the MnO_2_ electrodeposition time was systematically varied to maximize the electrochemical performance. According to the authors, CNFs acted not only as a dispersing agent for CNTs but also as a structural scaffold providing abundant hydroxyl and carboxyl groups that promoted homogeneous CNT distribution and offered active sites for MnO_2_ deposition. Moreover, the formation of interfacial C–O–Mn bonds was proposed to strengthen the adhesion between the MnO_2_ nanosheets and the CNF/CNT substrate, thereby improving structural stability. The optimized electrode, obtained with a CNF/CNT mass ratio of 4:6 and 40 min of MnO_2_ electrodeposition, achieves a specific capacitance of 1760 mF/cm^2^. After 10,000 cycles, the capacitance retention rate was 91.29%. The assembled flexible supercapacitor achieved an areal specific capacitance of 934 mF/cm^2^ and an energy density of 43.10 Wh/kg at a power density of 166.67 W/kg. Mechanical flexibility was qualitatively demonstrated through photographs of the device under bending, rolling, folding and wrapping conditions, while the corresponding CV curves showed negligible changes even at bending angles of 90° and 180°, indicating that mechanical deformation had little effect on the electrochemical performance. After 500 folding cycles at 180°, the device retained 93.6% of its initial capacitance.

The comparison between electrode-level and device-level performance also illustrates an important limitation in the current literature. The areal capacitance of the optimized electrode (1760 mF cm^−2^) is substantially higher than that of the assembled device (934 mF cm^−2^), highlighting the influence of measurement configuration and device architecture on the reported performance. Consequently, electrode-level metrics should not be directly compared with complete-device values, even when the same electrode material is used.

A further advance in cellulose-based composite electrodes was reported by Cheng et al. [49], who developed hierarchically core–shell structured aerogel electrodes composed of TEMPO-oxidized cellulose nanofibres (TOCNs), carbon nanotubes (CNTs), MnO_2_ and polyaniline (PANI). In this architecture, TOCNs acted simultaneously as a dispersant, structural template and hydrophilic bridge, promoting the homogeneous dispersion of CNTs and supporting the in situ polymerization of MnO_2_ and PANI to form a hierarchical core–shell conductive network. According to the authors, the interconnected porous architecture enhanced electrolyte accessibility, facilitated electron transport and provided mechanical robustness, while the synergistic contribution of MnO_2_ and PANI increased the pseudocapacitive charge storage. The resulting electrode exhibited a gravimetric capacitance of 334.8 F g^−1^. Beyond the electrode design, the authors assembled a flexible all-solid-state supercapacitor using the TCMP film together with a dynamically cross-linked cellulose nanofiber/PVA/borax hydrogel that imparted intrinsic self-healing capability. The device delivered an areal capacitance of 1108 mF cm^−2^ at 2 mA cm^−2^ and an areal energy density of 153.7 μWh cm^−2^ at 1101.7 µW cm^−2^ and retained 82% of its initial capacitance after 5000 charge–discharge cycles. Remarkably, it also maintained approximately 95% of its capacitance after 200 bending cycles and 90% after 10 cut/healing cycles, demonstrating that cellulose-based composite electrodes can be successfully integrated into multifunctional flexible energy-storage devices with autonomous self-repairing capability.

Although the enhanced performance is attributed to a synergistic contribution of MnO_2_ and PANI within the TOCN-based architecture, the multicomponent nature of the TCMP electrode makes it difficult to quantitatively disentangle the individual contributions and, particularly, the interactions between these components. The authors provide useful comparative controls by evaluating TC, TCM, and TCP alongside TCMP, which supports the contribution of MnO_2_ and PANI to the enhanced capacitance. However, such comparisons do not fully isolate the contribution of the natural polymer or quantify the extent to which the observed enhancement arises from synergistic interactions among TOCNs, CNTs, MnO_2_, and PANI. This illustrates a broader limitation across the literature: comparative controls are not always sufficient to distinguish individual component effects from emergent synergistic effects in multicomponent polymer–ceramic electrodes. Moreover, differences in capacitance normalization between electrode- and device-level measurements, such as F g^−1^ and mF cm^−2^, further complicate direct quantitative comparisons across studies.

More recently, attention has shifted towards advanced fabrication strategies capable of integrating natural polymers with thin ceramic coatings. A representative example, presented by Choudhary et al. [50], employed cellulose nanocrystals (CNCs) extracted from sugarcane bagasse, which act as a structural network on a conductive graphite substrate, while a thin MnO_2_ layer was deposited by radio-frequency magnetron sputtering. This combination takes advantage of the high surface area and structural stability of CNCs, the electrical conductivity of graphite, and the capacitive behavior of MnO_2_. Controlling the sputtering parameters enabled the deposition of a uniform and conformal coating of MnO_2_, while preserving the porous CNC structure. The formulation prepared with deposition at 150 °C exhibited the most favorable electrochemical performance among the conditions investigated. The symmetric device achieved an areal capacitance of 149 mF cm^−2^ within a 1.2 V potential window. The device also showed good cycling stability, retaining 85.27% of its initial capacitance after 15,000 cycles.

The authors compared CNC-, graphite-, and MnO_2_-containing formulations, highlighting the role of the conductive carbon framework and MnO_2_ in the overall performance. However, these comparisons do not fully disentangle the specific contribution of CNCs from the effects of the conductive network and MnO_2_ loading.

The authors also report that the cellulose-containing architecture remained cohesive, durable, and structurally stable during cycling, with minimal electrode cracking or delamination. Nevertheless, no systematic assessment of mechanical flexibility, such as bending or folding tests, was reported for the resulting device, limiting evaluation of its mechanical performance under repeated deformation.

Across the MnO_2_-based systems, the reviewed studies demonstrate a progressive diversification of the role of natural polymers, from flexible substrates to porous scaffolds, hydrogel matrices, structural modifiers, and multifunctional interfacial components. However, the comparison also reveals that the contribution of the natural polymer cannot be considered independently of the conductive network, ceramic loading, electrode architecture, and processing route. In several studies, improved performance is attributed to synergistic interactions between the polymer and electroactive ceramic, but the extent to which these contributions can be experimentally separated varies considerably among studies. Even when comparative formulations are included, they do not always fully isolate the individual roles of the natural polymer, ceramic phase, and conductive components.

**Figure 4:**
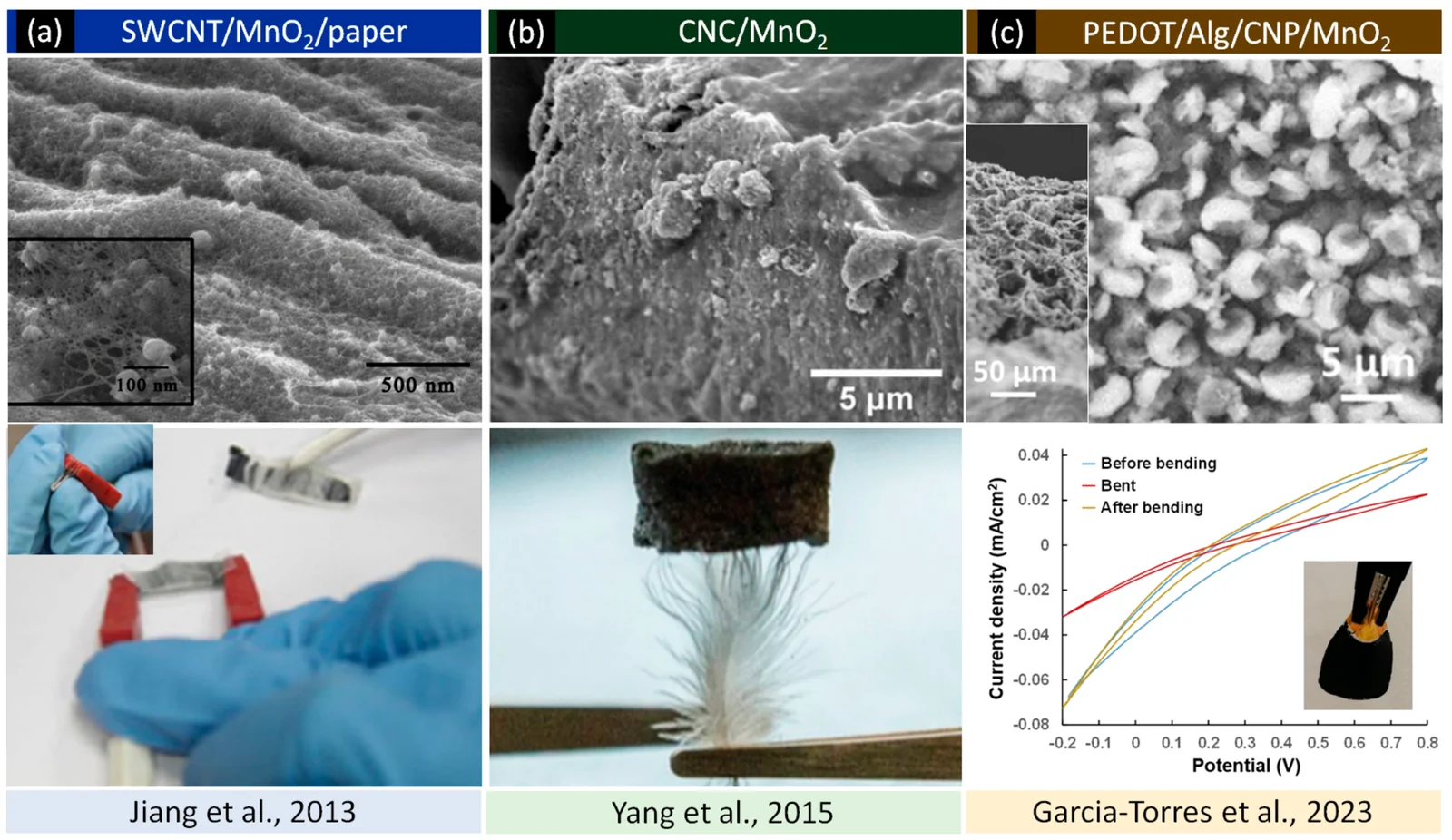
Representative SEM micrographs and demonstrations of device operation from selected studies on natural polymer–manganese-based ceramic composite electrodes for flexible energy-storage applications. (**a**) SWCNT/MnO_2_/paper electrode, showing a network of SWCNT bundles covering and interconnecting MnO_2_ particles (**top**), and rectangular flexible supercapacitors assembled from SWCNT/MnO_2_/paper electrodes evaluated under different bending conditions (**bottom**). (**b**) CNC–MnO_2_ electrode, showing the morphology of the composite aerogel (**top**), and the ultralight aerogel supported by a feather, highlighting its low density (**bottom**). (**c**) PEDOT/Alg/CNP/MnO_2_ electrode, showing a rough surface composed of aggregated round nanostructures, with the inset showing the porous and interconnected hydrogel structure (**top**), and cyclic voltammograms of the as-prepared, bent (completely folded), and recovered electrode after bending (**bottom**). Reproduced from Refs. [44,45,46] according to the copyright permissions or Creative Commons licenses of the original publications.

Representative morphological features and examples of device operation reported for selected MnO_2_-based natural polymer composite electrodes are presented in Figure 4. In addition, Table 2 summarizes the key electrochemical properties and flexibility-related mechanical performance of the formulations identified by the authors as optimal or best-performing in the studies discussed above.

#### 3.1.2. Iron-Based Electrodes

Xia et al. [51] developed a flexible CNF/MWCNT/RGO/Fe_3_O_4_ negative electrode for asymmetric supercapacitors by embedding Fe_3_O_4_ nanoparticles in situ into a three-dimensional porous hybrid aerogel composed of cellulose nanofibres (CNFs), multiwalled carbon nanotubes (MWCNTs) and reduced graphene oxide (RGO). The bottom-up assembly of the 1D CNFs and MWCNTs with 2D RGO produced a highly porous and hydrophilic conductive network, in which the CNFs promoted the dispersion of the carbon nanomaterials and contributed to the structural integrity and electrolyte accessibility, while the interconnected architecture facilitated electron transport and ion diffusion. At an optimized Fe^3+^ concentration of 0.08 mol L^−1^, corresponding to an Fe_3_O_4_ loading of 4.93 mg cm^−2^, the resulting electrode delivered an areal capacitance of 1193 mF cm^−2^ at 1 mA cm^−2^, with 98.1% capacitance retention after 3000 charge–discharge cycles. The electrode was subsequently coupled with a CF-CNF/MWCNT/MnO_2_ positive electrode to assemble a flexible quasi-solid-state asymmetric supercapacitor operating at up to 1.8 V. The device achieved a volumetric energy density of 2.35 mWh cm^−3^ and retained 94.7% of its initial capacitance after 5000 cycles. The device also maintained nearly unchanged CV profiles under different bending conditions, highlighting the mechanical flexibility and structural integrity of the cellulose-based architecture.

Following the development of cellulose-based flexible electrodes incorporating Fe_3_O_4_ and conductive carbon networks, Lv et al. (2018) [52] reported a different strategy based on a three-dimensional bacterial cellulose (BC) scaffold combined with Fe_3_O_4_ nanoparticles and polypyrrole (PPy). In this system, BC provided a flexible and mechanically robust fibrous framework, while Fe_3_O_4_ nanoparticles were deposited after in situ co-precipitation on the BC fibres and subsequently coated with a conductive PPy shell. The PPy layer was introduced to enhance the pseudocapacitance and acid stability, prevent volume expansion, and provide an electron-transport path in acidic electrolyte. The resulting BC/Fe_3_O_4_/PPy free-standing film exhibited a hierarchical fibrous structure, with Fe_3_O_4_ nanoparticles distributed along the BC fibers and a continuous PPy coating. Among the investigated formulations, the electrode prepared with 0.2 mol L^−1^ pyrrole exhibited the highest capacitance, reaching 5.43 F cm^−2^ at 1 mA cm^−2^ and 232.9 F g^−1^ at 1 A g^−1^. The incorporation of Fe_3_O_4_ increased the capacitance, while the PPy coating provided a conductive pathway and improved the electrical conductivity of the composite. The electrode also demonstrated good mechanical robustness, with a tensile strength of 11 MPa, and the authors consequently described the film as exhibiting “significant flexibility”. However, this claim should be interpreted with caution. The mechanical assessment was based on tensile testing of the free-standing film, rather than on electrochemical measurements performed under controlled and repeated mechanical deformation. In particular, no bending or folding tests coupled with capacitance retention measurements were reported, and no complete supercapacitor device was assembled and evaluated. Therefore, although the mechanical properties support the flexibility of the BC/Fe_3_O_4_@PPy film itself, the claimed potential for flexible supercapacitor applications is not directly demonstrated at the device level. This distinction is particularly relevant when assessing flexible energy-storage electrodes, for which mechanical flexibility and electrochemical stability under deformation are not necessarily equivalent. Cycling measurements revealed some dependence on the testing protocol: the capacitance retention reached 81.4% after 3500 cycles in the CV test, whereas the more demanding GCD test at 3 mA cm^−2^ resulted in approximately 50% retention, with an areal capacitance of 1.95 F cm^−2^ after 3500 cycles. Thus, the study demonstrates that a bacterial-cellulose scaffold can simultaneously provide structural integrity and a high-loading platform for Fe_3_O_4_/PPy pseudocapacitive electrodes, although the long-term stability is strongly dependent on the electrochemical testing conditions.

A comparison of these two Fe_3_O_4_-based architectures highlights that the role of the cellulose component is strongly dependent on the surrounding conductive network. In the CNF/MWCNT/RGO system, cellulose contributes to the formation and stabilization of the porous conductive scaffold, whereas in the BC/Fe_3_O_4_/PPy system the bacterial cellulose acts as a mechanically robust three-dimensional framework for Fe_3_O_4_ deposition. The substantially different capacitance values therefore cannot be interpreted simply as an effect of the cellulose type or Fe_3_O_4_ incorporation, since electrode composition, conductive additives, active-material loading, and testing configuration also differ. Importantly, the discrepancy between the reported cycling performances under CV and GCD conditions in the BC-based electrode demonstrates the need for caution when comparing cycling stability across studies.

While the previous study relied on a purpose-designed bacterial cellulose scaffold, Mendoza-Jiménez et al. [53] adopted a more waste-oriented and circular approach by using cellulose recovered from diaper waste as a flexible substrate for solid-state supercapacitors. The cellulose-containing diaper material was coated with graphene to form a conductive fibrous electrode and subsequently decorated with PbFe_11_CrO_19_ (PbFeCr) ferrite nanorods. The interconnected cellulose fiber network provided a porous structure with free spaces that facilitated electrolyte penetration, while graphene established a conductive network and the PbFeCr ferrite introduced additional redox-active sites for charge storage, also enhancing the electrochemical stability and capacitance. The resulting flexible symmetric device exhibited a gravimetric capacitance of 1894.8 F g^−1^ and an energy density of 164.4 Wh kg^−1^ at 1 A g^−1^, substantially exceeding the performance of the graphene-coated diaper reference device. The improved performance was associated with the fibrous electrode morphology, the rod-like PbFeCr nanostructures, and the additional redox centres associated with Fe^2+^/Fe^3+^ and Cr^3+^/Cr^6+^ species. The device retained 90.3% of its initial capacitance after 2000 charge–discharge cycles. Mechanical flexibility was further evaluated by bending the device at different radii of curvature. At the maximum tested curvature, corresponding to a radius of 2.8 cm, the DFG/PbFeCr-SC retained 88.2% of its initial capacitance. However, bending increased the electrical resistance, suggesting the formation of cracks or damage within the electrodes. Overall, this work demonstrates the potential of waste-derived cellulose-containing substrates for flexible energy-storage devices, while highlighting the performance enhancement achieved through the incorporation of redox-active ferrite nanostructures.

However, the very high capacitance reported for this system should be interpreted with caution when compared with the other Fe-based electrodes, as the electrode incorporates several electrochemically active components, including graphene and PbFeCr ferrite, and the specific contribution of the recovered cellulose cannot be isolated from the reported data. Thus, the improvement should not be attributed solely to the incorporation of the ferrite or to the cellulose-derived fibrous morphology. The increase in electrical resistance observed upon bending, together with the decrease in capacitance retention to 88.2% at the maximum tested curvature, further indicates that morphological flexibility does not necessarily translate into fully robust electrochemical performance under deformation.

More recently, Zhao et al. [54] developed a flexible electrode based on cellulose nanofibres (CNFs), multiwalled carbon nanotubes (MWCNTs) and Fe_3_O_4_ nanoparticles. This study employed CNFs as a structural component of a free-standing nanopaper and combined them with MWCNTs to establish a three-dimensional conductive network. Fe_3_O_4_ nanoparticles were first grown in situ on the surface of MWCNTs, producing MWCNT@Fe_3_O_4_ nanocomposites, which were subsequently incorporated into the CNF/MWCNT nanopaper. The resulting electrode, denoted NFT@Fe, therefore combined the structural and film-forming properties of CNFs with the electrical conductivity of MWCNTs and the pseudocapacitive and magnetic properties of Fe_3_O_4_. A series of Fe_3_O_4_/MWCNT/CNF composite nanopapers was prepared by varying the relative amounts of Fe_3_O_4_-decorated MWCNTs, pristine MWCNTs and CNFs. The materials were denoted NFT@Fe-xyz, where xyz represents the corresponding mass ratio of the three components. An initial series varied the relative amounts of Fe_3_O_4_-decorated MWCNTs and CNFs, while a second series maintained the CNF content constant and progressively replaced Fe_3_O_4_-decorated MWCNTs with pristine MWCNTs. A CNF/MWCNT nanopaper without Fe_3_O_4_ was also prepared as a reference. The electrochemical evaluation of the second series identified NFT-712, corresponding to a 7:1:2 mass ratio of Fe_3_O_4_-decorated MWCNTs, pristine MWCNTs and CNFs, as the optimal composition. This formulation exhibited a specific capacitance of 229.9 F g^−1^, corresponding to an areal capacitance of 735.68 mF cm^−2^, at 5 mV s^−1^. The optimized nanopaper was subsequently used as the negative electrode in a flexible quasi-solid-state asymmetric supercapacitor, with an unmodified CNF/MWCNT nanopaper (NFT) as the positive electrode and a CMC/Li_2_SO_4_ gel electrolyte. The assembled NFT@Fe-712//NFT device operated within a 1.6 V potential window and achieved a specific capacitance of 107.0 F g^−1^ at 0.5 A g^−1^, equivalent to 2.94 F cm^−2^. It delivered an energy density of 38.0 Wh kg^−1^ at a power density of 0.405 kW kg^−1^ and retained an energy density of 18.8 Wh kg^−1^ at a power density of 42.2 kW kg^−1^. More than 90% of the initial capacitance was retained after 5000 charge–discharge cycles. The device also demonstrated pronounced mechanical flexibility. Electrochemical measurements were performed under different bending angles, and after five cycles at 180° bending, the device retained 98% of its capacitance. Beyond its electrochemical and mechanical performance, the device could be disassembled, allowing the Fe_3_O_4_-containing electrode material to be magnetically separated and recovered. The material subsequently exhibited almost complete degradation after 50 days in soil, highlighting an additional end-of-life advantage compared with conventional flexible supercapacitor electrodes.

The systematic variation in the Fe_3_O_4_-decorated MWCNT, pristine MWCNT, and CNF contents provides a more informative basis for identifying composition–performance relationships than studies based on a single optimized formulation. In particular, the use of an Fe_3_O_4_-free CNF/MWCNT nanopaper as a reference provides a useful basis for assessing the contribution of the ferrite phase relative to the conductive cellulose-based scaffold. Nevertheless, the optimized composition reflects a balance between pseudocapacitive Fe_3_O_4_ loading and the conductive and structural functions provided by the MWCNT/CNF network, indicating that increasing the amount of electroactive ceramic is not necessarily beneficial without maintaining an adequate conductive network.

Building on their previous Fe_3_O_4_/MWCNT/CNF nanopaper design, Zhao et al. [55] extended this approach by employing two different ferrite-based nanocomposites as the negative and positive electrodes of a flexible asymmetric supercapacitor. Fe_3_O_4_ and MnFe_2_O_4_ nanoparticles were grown in situ on MWCNTs through a one-pot thermal decomposition process, while CNFs were subsequently used to disperse the resulting nanocomposites in water and promote the formation of stable electrode inks. The negative and positive nanopaper electrodes were produced by incorporating Fe_3_O_4_-decorated and MnFe_2_O_4_-decorated MWCNTs, respectively, into the CNF network. The authors denoted these materials NFT@Fe and NFT@MnFe, respectively. The two electrode inks were then patterned into an interdigital architecture by mask-assisted vacuum filtration, followed by the incorporation of a CMC/Li_2_SO_4_ hydrogel electrolyte to assemble a planar flexible asymmetric supercapacitor. Charge balancing between the two electrodes was achieved by adjusting the mass ratio of the positive and negative materials to 1:1.37. The electrochemical performance was subsequently tuned by varying the volume of both electrode inks from 50 to 250 μL, thereby increasing the electrode thickness and the loading of electrochemically active materials. Among the investigated devices, the formulation prepared with 250 μL of each electrode ink, designated NFT@Fe//NFT@MnFe-250, exhibited the highest areal capacitance, reaching 309.8 mF cm^−2^ at 0.5 mA cm^−2^. The device also delivered an areal energy density of 110.2 μWh cm^−2^ and retained more than 95% of its initial capacitance after 5000 charge–discharge cycles at 5 mA cm^−2^. The device also demonstrated pronounced mechanical flexibility, retaining 96.3% of its initial capacitance at the maximum tested bending angle of 180°. The same electrode configuration was also used to construct a stacked interdigital device, which increased the areal energy density to 273.8 μWh cm^−2^ at a power density of 0.47 mW cm^−2^.

This study further illustrates the strong influence of electrode architecture and active-material loading on the reported performance. Increasing the ink volume simultaneously increases electrode thickness, active-material loading, and the development of the three-dimensional conductive network, making it difficult to attribute the observed improvement to any single factor. The higher areal energy density obtained with the stacked architecture further demonstrates that device architecture and active-material loading can substantially influence reported performance metrics, beyond the intrinsic properties of the polymer–ceramic composite. Therefore, comparisons based exclusively on capacitance or energy density may be misleading unless electrode thickness, active-material loading, and device configuration are considered simultaneously.

Concluding this group of iron-based composite electrodes, a simpler Fe_3_O_4_–CNF architecture was reported, by Rabani et al. [56], for the fabrication of a flexible solid-state symmetric supercapacitor. Four Fe_3_O_4_/CNF formulations were prepared by varying the Fe_3_O_4_ precursor concentration and synthesis temperature. While Fe_3_O_4_@CNF1 and Fe_3_O_4_@CNF2, with Fe_3_O_4_:CNF ratios of 2:1 and 1.5:1, respectively, synthesized at 25 °C, showed pronounced nanoparticle aggregation, Fe_3_O_4_@CNF3 and Fe_3_O_4_@CNF4 were prepared using a 1:1 ratio at 60 and 100 °C, respectively. The Fe_3_O_4_@CNF4 formulation exhibited the most uniform nanoparticle distribution and was therefore selected for device fabrication. The optimized Fe_3_O_4_@CNF4 paper was subsequently carbonized at 200 °C under a H_2_/Ar atmosphere for 20 min prior to device assembly. This low-temperature treatment improved both its mechanical and electrochemical properties, increasing the mechanical strength by approximately 11% while enhancing the capacitive response through increased carbon content and electronic conductivity. The carbonized Fe_3_O_4_@CNF4 electrode was then assembled into a symmetric solid-state supercapacitor using a PVA–KOH gel electrolyte. The resulting device delivered a specific capacitance of 188.3 F g^−1^ at 0.5 A g^−1^, together with a maximum energy density of 33.9 Wh kg^−1^ at a power density of 0.54 kW kg^−1^. It also exhibited excellent cycling stability, retaining 94% of its initial capacitance after 8000 charge–discharge cycles. Mechanical flexibility was further supported by the properties of the optimized Fe_3_O_4_@CNF4 paper, which exhibited a tensile strength of 62 MPa and an elongation at break of 79.2%. In addition, CV measurements performed at bending angles from 0° to 180° showed that the electrochemical profiles remained almost unaffected, indicating stable electrochemical performance under substantial mechanical deformation. Beyond its electrochemical function, the spent Fe_3_O_4_@CNF4 electrode was subsequently disassembled and reutilized as a photocatalyst for the degradation of an organic dye under visible-light irradiation, illustrating an additional route for the post-use valorization of the electrode material.

Compared with the previous Fe_3_O_4_/cellulose systems, this study introduces an additional processing variable through low-temperature carbonization of the CNF-based electrode. Consequently, the resulting electrochemical response reflects not only the Fe_3_O_4_–CNF interaction but also structural and electronic changes induced by carbon formation during thermal treatment. This distinction is important when comparing the performance of carbonized and non-carbonized cellulose-based electrodes, since the processing route partially transforms the chemical and electronic characteristics of the original biopolymer and, consequently, its role within the electrode architecture.

Overall, the Fe-based studies demonstrate that the performance of cellulose–ferrite electrodes is governed by a complex interplay between the cellulose-derived scaffold, ferrite chemistry and loading, conductive additives, electrode architecture, and processing conditions. Cellulose can contribute to structural integrity, flexibility, dispersion, and electrolyte accessibility, while Fe-based phases provide redox-active sites; however, the relative importance of these contributions varies substantially between architectures and is not always experimentally separable. The comparison also shows that higher electrochemical performance is not necessarily associated with higher ceramic loading, as excessive active-material incorporation may compromise conductive pathways, ion transport, or structural integrity. Moreover, mechanical flexibility demonstrated at the material or electrode level does not necessarily translate into electrochemical robustness under deformation at the device level. Differences in electrode loading, thickness, testing configuration, and device architecture further complicate direct quantitative comparisons across studies.

**Figure 5:**
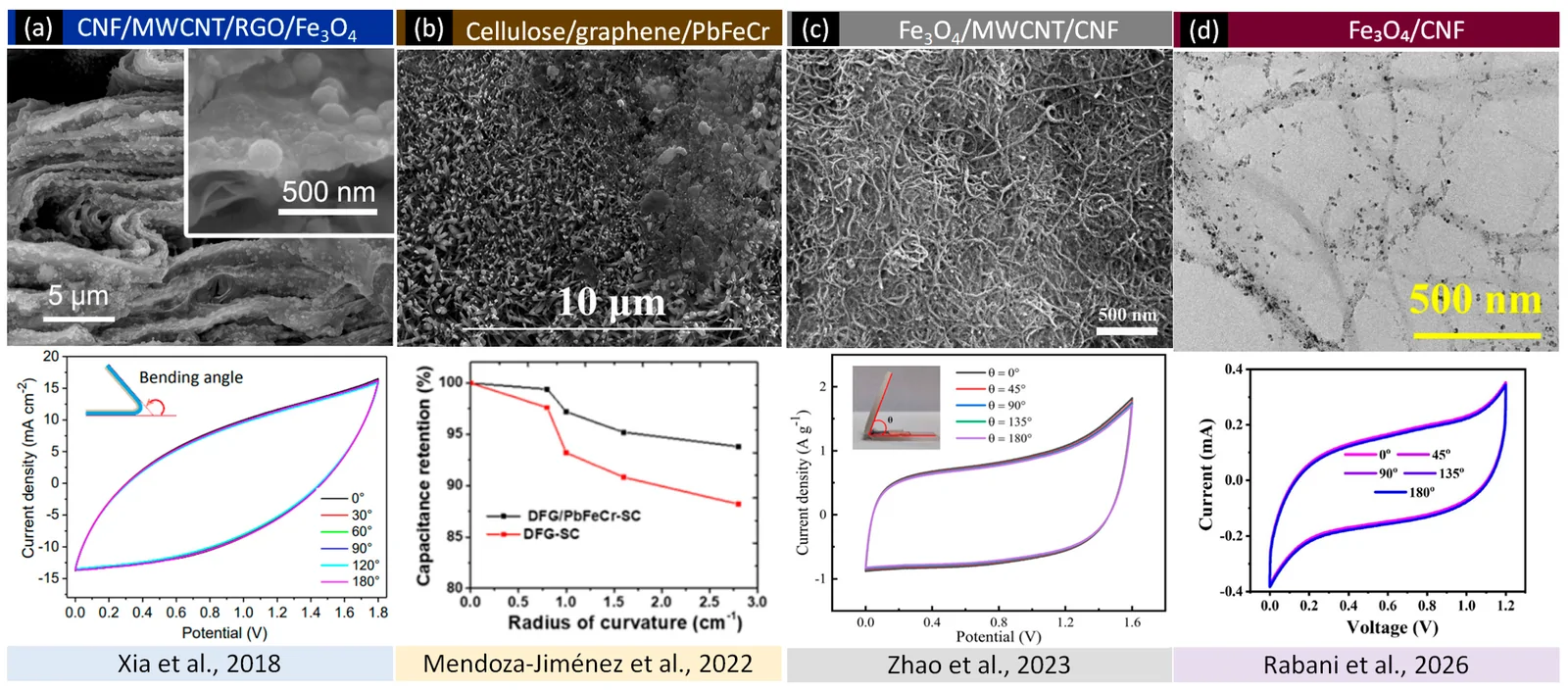
Representative SEM micrographs and demonstrations of device operation from selected studies on natural polymer–iron-based ceramic composite electrodes for flexible energy-storage applications. (**a**) CNF/MWCNT/RGO/Fe_3_O_4_ negative electrode, showing multiple layers of sandwich-like structures, with Fe_3_O_4_ nanoparticles uniformly embedded between the CNF/MWCNT/RGO layers (**top**), and CV curves of the flexible asymmetric supercapacitor at different bending angles at a scan rate of 25 mV s^−1^ (**bottom**). (**b**) Cellulose/graphene/PbFe_11_CrO_19_ electrode, showing the formation of the graphene/ferrite composite on the diaper-derived cellulose surface, with approximately 68% of the cellulose estimated to be coated with graphene/PbFeCr nanoparticles (**top**), and capacitance retention as a function of the radius of curvature (**bottom**). (**c**) Fe_3_O_4_/MWCNT/CNF electrode, showing an entangled fibrous structure forming a three-dimensional network (**top**), and CV curves obtained at various bending angles at 5 mV s^−1^ (**bottom**). (**d**) Fe_3_O_4_/CNF electrode, showing the Fe_3_O_4_ nanosized particles uniformly dispersed on the surface of CNFs in the Fe_3_O_4_@CNF4 sample (**top**), and CV profiles recorded at different bending angles (**bottom**). Reproduced from Refs. [51,53,54,56] according to the copyright permissions.

Representative SEM micrographs and demonstrations of device operation reported for selected natural polymer–iron-based ceramic composite electrodes are presented in Figure 5. Table 3 summarizes the key electrochemical properties and flexibility-related mechanical performance of the formulations identified by the authors as optimal or best-performing in the studies discussed above.

#### 3.1.3. Tin-Based Electrodes

Turning to SnO_2_-based composite electrodes, Liu et al. [57] incorporated SnO_2_ nanoparticles and graphene oxide (GO) flakes directly into a bacterial nanocellulose (BNC) matrix during its bacteria-mediated growth. After purification, the embedded GO was chemically reduced to rGO, and the resulting SnO_2_/rGO/BNC film was subsequently coated with the conductive polymer PEDOT:PSS to obtain a flexible hybrid electrode. This fabrication strategy generated a layered structure in which SnO_2_ nanoparticles were embedded within the rGO/BNC network, while the PEDOT:PSS coating provided additional electrical conductivity.

The incorporation of SnO_2_ substantially enhanced the electrochemical performance, with the PEDOT:PSS/SnO_2_/rGO/BNC electrode achieving a specific capacitance of 445 F g^−1^ at 2 A g^−1^ and retaining 84.1% of its initial capacitance after 2500 charge–discharge cycles. The authors attributed the enhanced capacitance to the reversible faradaic processes associated with SnO_2_, with pseudocapacitive contributions accounting for approximately 69% of the total capacitance.

However, although the comparative formulations provide relatively strong evidence for the contribution of SnO_2_ to the overall capacitance, the specific contribution of the cellulose-based BNC scaffold remains difficult to isolate because the optimized electrode contains several electrochemically relevant components, including rGO and PEDOT:PSS, in addition to BNC. The reported pseudocapacitive contribution of approximately 69% supports the importance of SnO_2_-related faradaic processes, but does not, by itself, establish the extent to which the BNC matrix contributes to the measured performance or how it interacts with the conductive and pseudocapacitive components. More broadly, this highlights the difficulty of quantitatively attributing performance improvements to individual components in highly integrated multicomponent electrodes.

Two identical PEDOT:PSS/SnO_2_/rGO/BNC electrodes were subsequently assembled into a flexible solid-state supercapacitor, using a BNC separator impregnated with a PVA–H_2_SO_4_ gel electrolyte. The device exhibited a specific capacitance of 603 F g^−1^ at 0.5 A g^−1^ and retained 89.8% of its initial capacitance after 2500 cycles. Flexibility was further demonstrated through CV measurements at different bending angles, which showed stable electrochemical behaviour under deformation.

The difference between the reported electrode-level and device-level specific capacitance values also illustrates the difficulty of comparing electrochemical metrics obtained at different stages of device development. Although both values are reported on a gravimetric basis, electrode-level and full-device measurements involve different electrode configurations and operating conditions, and may differ in the mass considered for normalization. Consequently, these values should not be interpreted as directly equivalent indicators of intrinsic material performance.

Following the BNC-based SnO_2_ composite described above, Tan et al. [58] adopted a different cellulose-based architecture, using cellulose nanofibres (CNFs) to fabricate a three-dimensional porous aerogel incorporating reduced graphene oxide (RGO) and SnO_2_. In this case, hydroquinone was used during the hydrothermal reduction process as both a reducing and functionalizing agent, after which the RGO/SnO_2_ phase was combined with CNFs, freeze-dried to form an aerogel and subsequently compressed into a flexible electrode film. The initially described fabrication procedure yielded the hydroquinone-functionalized CNF/RGO/SnO_2_ composite aerogel, denoted F-CRA. For comparison, two additional formulations, CRA and m-CRA, were subsequently prepared using modified preparation routes. Among the three formulations, F-CRA exhibited the highest electrochemical performance.

Its interconnected porous architecture, formed by crisscrossed CNF and RGO structures, provided microchannels that facilitated electrolyte-ion transport. The F-CRA electrode achieved an areal capacitance of 4.3 F cm^−2^ at 1 mA cm^−2^. The enhanced capacitance relative to CRA and m-CRA was mainly associated with the hydroquinone-mediated reduction of graphene. However, cycling stability remained limited, with 60.47% capacitance retention after 2000 cycles at 10 mA cm^−2^, which the authors attributed to the progressive consumption of the hydroquinone molecules contributing to the capacitance.

The limited cycling stability further illustrates this trade-off: the same hydroquinone-derived contribution that enhances the initial electrochemical response may compromise long-term stability as the electroactive species are progressively consumed. Thus, high initial capacitance does not necessarily translate into superior overall electrode performance. Although no mechanical characterization or electrochemical bending tests were reported, this study highlights the potential of CNFs as a structural scaffold for constructing porous SnO_2_-containing hybrid electrodes with high areal capacitance. However, the flexibility of the electrode cannot be quantitatively assessed or directly compared with mechanically and electrochemically validated systems discussed elsewhere in this review. The comparison between F-CRA, CRA, and m-CRA provides useful evidence that processing-induced changes can be as important as composition in determining electrochemical performance. In particular, the superior performance of F-CRA was mainly associated with hydroquinone-mediated graphene reduction rather than with an isolated effect of SnO_2_ or CNF. This distinction is important because the three formulations differ in both composition and processing history, making it difficult to attribute the performance enhancement exclusively to the cellulose–SnO_2_ combination.

Building on the use of cellulose/rGO/SnO_2_ composites described above, Garino et al. [59] employed microfibrillated cellulose (MFC) to fabricate self-standing composite membranes containing SnO_2_-decorated reduced graphene oxide (rGO). The rGO/SnO_2_ nanocomposite was synthesized through a microwave-assisted hydrothermal process, which simultaneously reduced GO and promoted the formation of SnO_2_ nanoparticles on the graphene surface. The resulting nanocomposite was subsequently mixed with an aqueous MFC suspension and cast into self-standing membranes. Three compositions containing 33, 50, and 66 wt% rGO/SnO_2_ were investigated, with the MFC fraction decreasing accordingly. The 66 wt% formulation was selected for supercapacitor assembly based on its electrochemical performance, whereas lower and higher rGO/SnO_2_ contents resulted in insufficient conductivity and mechanical stability, respectively.

The systematic variation in the rGO/SnO_2_ content provides evidence for an important composition–property trade-off. Increasing the rGO/SnO_2_ fraction enhances the electrical and electrochemical response within the investigated composition range, whereas excessive incorporation compromises mechanical stability and limits the practical composition window. Conversely, higher MFC contents reduce the conductive fraction and result in lower electrical conductivity. Thus, the results indicate that the natural polymer is not merely a passive flexible substrate; its fraction must be balanced against the conductive and electrochemically active phases to maintain both mechanical integrity and charge transport.

The device achieved a maximum specific capacitance of 53 F g^−1^ at a scan rate of 10 mV s^−1^ and retained more than 90% of its initial capacitance after 1000 cycles. In contrast with the previous study, the flexibility of the present composite was experimentally evaluated, with the self-standing membranes reported to resist bending, with Young’s modulus values above 200 MPa and tensile strength ranging from 0.9 to 4.2 MPa depending on composition. Furthermore, the supercapacitor retained more than 80% of its capacitance under high bending angles, demonstrating its ability to withstand mechanical deformation while maintaining electrochemical performance.

This study therefore provides stronger evidence of practical flexibility than studies in which flexibility is inferred only from visual bending or qualitative descriptions. The combination of quantitative mechanical characterization and electrochemical testing under deformation allows a more meaningful assessment of the relationship between mechanical integrity and electrochemical stability. Nevertheless, the retention of more than 80% of the capacitance under bending demonstrates that the electrode can maintain electrochemical functionality while undergoing mechanical deformation, although the study does not quantitatively establish a direct relationship between mechanical properties and electrochemical retention.

Beyond its application in supercapacitors, the rGO/SnO_2_/MFC composite was also evaluated for piezoresistive sensing and as a catalyst for the oxygen reduction reaction, highlighting its multifunctional potential.

As a final example within the SnO_2_-based systems, Letchumanan et al. [60] explored a simpler MFC/SnO_2_ architecture, avoiding the use of additional conductive carbon nanomaterials such as rGO. In this approach, self-standing MFC thin films were first prepared and subsequently coated with a SnO_2_–MFC nanocomposite obtained by hydrothermal treatment. Three SnO_2_ loadings, namely 4, 8, and 12 wt%, were investigated, with the SnO_2_–MFC slurry being deposited onto the MFC films by dip coating. The resulting hybrid films retained their self-standing and flexible character after coating, while the hydrothermal treatment produced a flower-like surface morphology. Increasing the SnO_2_ content enhanced the capacitive response, with the highest SnO_2_ loading exhibiting the best electrochemical performance. The corresponding electrode achieved a specific capacitance of 486.38 F g^−1^ at 20 mV s^−1^ and 225.88 F g^−1^ at 100 mV s^−1^, with 95% capacitance retention after only 40 cycles at 100 mV s^−1^, thus providing limited evidence of its long-term cycling stability. Although the films were visually shown to be flexible and proposed as foldable electrodes, no quantitative mechanical characterisation or electrochemical evaluation under bending was reported. Moreover, the electrochemical measurements were performed in a three-electrode configuration; therefore, energy and power densities for a complete supercapacitor device were not provided.

The high specific capacitance reported for the 12 wt% SnO_2_ electrode should, however, be interpreted in the context of the testing configuration and limited cycling data. The measurements were conducted in a three-electrode configuration, which does not allow direct comparison with full-device performance, while only 40 CV cycles were reported. Moreover, although the films were described as foldable, electrochemical retention under bending was not quantified. Consequently, the high initial capacitance demonstrates the electrochemical potential of the SnO_2_/MFC architecture but provides insufficient evidence to establish its long-term suitability for durable flexible energy-storage devices.

Overall, the SnO_2_-based studies demonstrate that the performance of cellulose-based flexible electrodes is governed by the interplay between the cellulose scaffold, SnO_2_ loading and distribution, conductive carbon phases, additional redox-active polymers, and processing conditions. Across these systems, cellulose can contribute to structural integrity, porosity, electrolyte accessibility, and mechanical flexibility, whereas SnO_2_ provides electrochemically active sites. However, the magnitude of the reported improvement cannot always be attributed specifically to SnO_2_, particularly in multicomponent electrodes containing rGO, PEDOT:PSS, or other electroactive species. The studies also reveal a recurrent composition–performance trade-off. Increasing the conductive or electrochemically active fraction can improve charge storage and electrical conductivity within a given composition range, but excessive incorporation may compromise mechanical integrity or long-term stability. Conversely, increasing the cellulose fraction can improve structural robustness while reducing electrical conductivity. The optimum composition is therefore architecture- and processing-dependent rather than universally defined. Importantly, the comparison also highlights that reported electrochemical performance depends strongly on the testing configuration and device architecture. High electrode-level capacitance obtained in three-electrode measurements cannot be directly equated with complete-device performance, while visual or qualitative demonstrations of flexibility do not provide the same level of evidence as quantitative mechanical characterization combined with electrochemical testing under deformation. These differences should therefore be considered when assessing the practical potential of SnO_2_–cellulose electrodes for flexible energy-storage applications.

**Figure 6:**
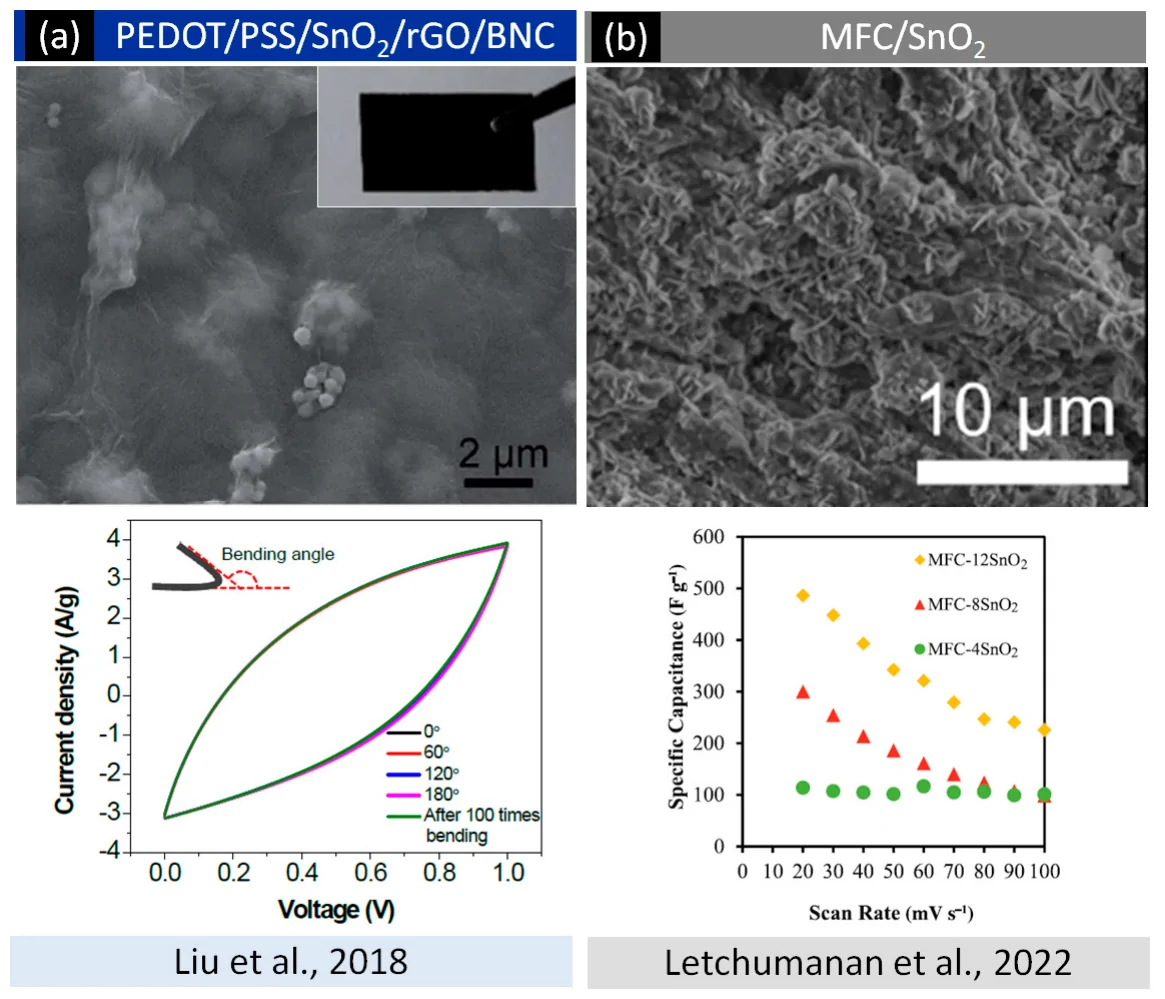
Representative SEM micrographs and demonstrations of device operation from selected studies on natural polymer–tin-based ceramic composite electrodes for flexible energy-storage applications. (**a**) PEDOT:PSS/SnO_2_/rGO/BNC electrode, showing SnO_2_ nanoparticles embedded within the GO/BNC layers, with the inset showing a digital image of the SnO_2_/GO/BNC film (**top**), and CV curves of the supercapacitor recorded at different bending angles at a scan rate of 100 mV s^−1^ (**bottom**). (**b**) MFC/SnO_2_ electrode, showing the SnO_2_–cellulose nanocomposite attached to the MFC thin film, forming a flower-like surface structure (**top**), and the specific capacitance calculated from cyclic voltammetry data for each sample (**bottom**). Reproduced from Refs. [57,60] according to the copyright permissions.

Representative morphological features and demonstrations of device operation from selected SnO_2_-based natural polymer composite electrodes are presented in Figure 6. Table 4 summarizes the key electrochemical properties and flexibility-related mechanical performance of the formulations identified by the authors as optimal or best-performing in the studies discussed above.

#### 3.1.4. Cobalt-Based Electrodes

Moving to Co_3_O_4_-based systems, Rabani et al. [61] developed a one-dimensional Co_3_O_4_/cellulose nanofiber (CNF) hybrid electrode through a sol–gel approach, in which Co_3_O_4_ nanoparticles were grown directly onto the CNF surface. Three Co_3_O_4_:CNF precursor ratios, namely 0.5:1, 1:1, and 1.5:1, corresponding to Co_3_O_4_@CNF1, Co_3_O_4_@CNF2, and Co_3_O_4_@CNF3, respectively, were investigated. Increasing the Co_3_O_4_ content improved the electrochemical response up to the 1:1 ratio, whereas the higher loading promoted nanoparticle aggregation, reducing the accessible surface area and electrical conductivity.

This composition-dependent behaviour highlights that increasing the ceramic fraction does not necessarily translate into improved electrochemical performance. Instead, the results indicate an optimum Co_3_O_4_:CNF ratio, at which sufficient electroactive material is combined with uniform nanoparticle dispersion and effective electrical contact with the CNF scaffold. The deterioration observed at higher Co_3_O_4_ loading, associated with nanoparticle aggregation and reduced electrical conductivity, provides direct evidence of a trade-off between electroactive-material loading and the accessibility and electrical connectivity of the hybrid structure.

Among the investigated formulations, Co_3_O_4_@CNF2, with a Co_3_O_4_:CNF ratio of 1:1, showed the most favourable electrochemical performance, attributed to the uniform distribution of approximately 5 nm Co_3_O_4_ nanoparticles along the CNF surface. The comparison among the three compositions further suggests that nanoparticle dispersion is an important performance descriptor, rather than Co_3_O_4_ content alone. At the optimum composition, the CNF network provides sufficient interfacial area and structural separation to maintain accessible Co_3_O_4_ active sites, whereas excessive loading favours nanoparticle aggregation. This observation supports the importance of controlling the polymer–ceramic interface rather than simply maximizing the amount of electroactive ceramic.

In a three-electrode configuration, this hybrid electrode delivered a specific capacitance of 789.9 F g^−1^ at 1 A g^−1^, retaining 346.3 F g^−1^ at 5 A g^−1^. The optimized material was subsequently assembled into a symmetric supercapacitor, in which two identical Co_3_O_4_@CNF electrodes were evaluated using an aqueous KOH electrolyte. The device achieved a maximum energy density of 26.7 Wh kg^−1^ at a power density of 0.9 kW kg^−1^ and retained ~94% of its initial capacitance after 5000 charge/discharge cycles. For flexible device fabrication, the Co_3_O_4_@CNF material was vacuum-filtered into a paper-like electrode and subjected to a low-temperature treatment at 200 °C under a H_2_/Ar atmosphere before assembly into a symmetric solid-state supercapacitor using a PVA–KOH gel electrolyte. The resulting flexible device delivered a specific capacitance of ~80 F g^−1^ at 1 A g^−1^, together with a maximum energy density of 10 Wh kg^−1^ at a power density of 0.9 kW kg^−1^. Its electrochemical response remained essentially unchanged under bending at angles up to 120° and after 50 random bending cycles.

A substantial decrease in specific capacitance is observed when moving from the three-electrode configuration to the flexible solid-state device, from 789.9 to approximately 80 F g^−1^. This difference illustrates the strong influence of electrode configuration, electrolyte environment, device architecture, and potentially mass-normalization procedures on the measured electrochemical response. Therefore, the high capacitance obtained at the electrode level should not be directly interpreted as representative of the performance achievable in a practical flexible device. The corresponding reduction in energy density from the aqueous symmetric device to the flexible solid-state configuration, from 26.7 to 10 Wh kg^−1^, further shows that the transition to a flexible solid-state architecture was accompanied by a reduction in the reported energy density. However, this difference cannot be attributed solely to solid-state operation or mechanical integration, since the flexible device also involved a different electrode-processing route, including low-temperature thermal treatment.

Following the Co_3_O_4_/CNF system described above, Xiao et al. [62] adopted a different strategy to fabricate free-standing Co_3_O_4_-based electrodes, used as both a flexible substrate and binder for porous Co_3_O_4_ polyhedra (PCP). The Co_3_O_4_ active material was obtained through a two-step calcination of ZIF-67, with PCP-400 selected based on its preserved polyhedral morphology and the highest specific surface area among the investigated calcination conditions. The electrodes were fabricated through a water-based paper-making process. NFC, porous Co_3_O_4_ polyhedron (PCP), and acetylene black (AB) acted as building skeleton and flexible substrate, active material, and conductive additive, respectively. Four formulations containing 50, 60, 70, and 80 wt% PCP-400 were investigated and denoted NPC-50, NPC-60, NPC-70, and NPC-80, respectively. Among these formulations, NPC-60 exhibited the highest areal capacitance, reaching 594.8 mF cm^−2^ at 5 mV s^−1^, indicating that an intermediate PCP loading provided a more favourable balance between electrochemical activity and structural integrity.

This systematic variation in PCP content provides further evidence that the relationship between ceramic loading and electrochemical performance is non-linear. The optimum at 60 wt% suggests that the NFC scaffold is required not only to impart flexibility but also to maintain a favourable balance between active-material loading and structural integrity. At higher PCP contents, the reduced relative NFC fraction can compromise the integrity of the free-standing electrode, as evidenced by active-material detachment from the NFC substrate in the NPC-80 film. Thus, the natural polymer fraction should be regarded as a functional design parameter rather than merely as a flexible substrate.

NPC-60 was subsequently evaluated in a symmetric supercapacitor configuration, delivering a specific capacitance of 52.74 F g^−1^ at 1 A g^−1^, with a maximum energy density of 18.75 Wh kg^−1^ and a power density of 8.00 kW kg^−1^. However, its cycling stability was relatively limited, retaining only 64% of its initial capacitance after 2000 cycles, which the authors attributed to the moderate conductivity of the porous Co_3_O_4_ component. The NPC-60 film was also visually demonstrated to withstand bending and rolling, confirming the flexibility and foldability of the free-standing electrode; however, no quantitative mechanical characterization or electrochemical evaluation under repeated bending was reported. The evidence for flexibility is therefore less extensively validated than that reported for the Co_3_O_4_@CNF system, where electrochemical behaviour was quantified under bending up to 120° and after repeated deformation. This difference illustrates the need to distinguish between qualitative demonstrations of foldability and experimentally validated mechanical–electrochemical durability when assessing flexible energy-storage materials.

While the study demonstrates the potential of nanocellulose to enable flexible and self-supporting Co_3_O_4_ electrodes, the limited cycling stability remains an important limitation of the proposed system.

Although the authors associated the limited cycling stability with the moderate conductivity of the porous Co_3_O_4_ component, the available data do not allow this effect to be isolated from other possible factors, such as electrode architecture, interfacial stability, and structural evolution during cycling. This illustrates a recurring limitation in the literature, in which degradation mechanisms are often attributed to a single material component despite the multicomponent nature of the electrode.

Overall, the Co_3_O_4_-based studies demonstrate that the performance of cellulose–ceramic electrodes is strongly governed by the balance between electroactive material loading, nanoparticle dispersion, electrical connectivity, and structural integrity. Both studies identify an intermediate ceramic loading as favourable, indicating that maximizing Co_3_O_4_ content is not an effective general strategy. Instead, the cellulose-based scaffold plays an active role in maintaining dispersion, accessible surface area, mechanical integrity, and transport pathways.

The comparison also highlights the importance of distinguishing electrode-level performance from practical device performance. In the Co_3_O_4_@CNF system, the specific capacitance decreased substantially when transitioning from a three-electrode measurement to the flexible solid-state device, while the energy density was also reduced. This difference demonstrates that electrode configuration, electrolyte environment, device architecture, and processing conditions can substantially affect the reported performance and should therefore be considered when assessing the practical relevance of electrode-level measurements.

**Figure 7:**
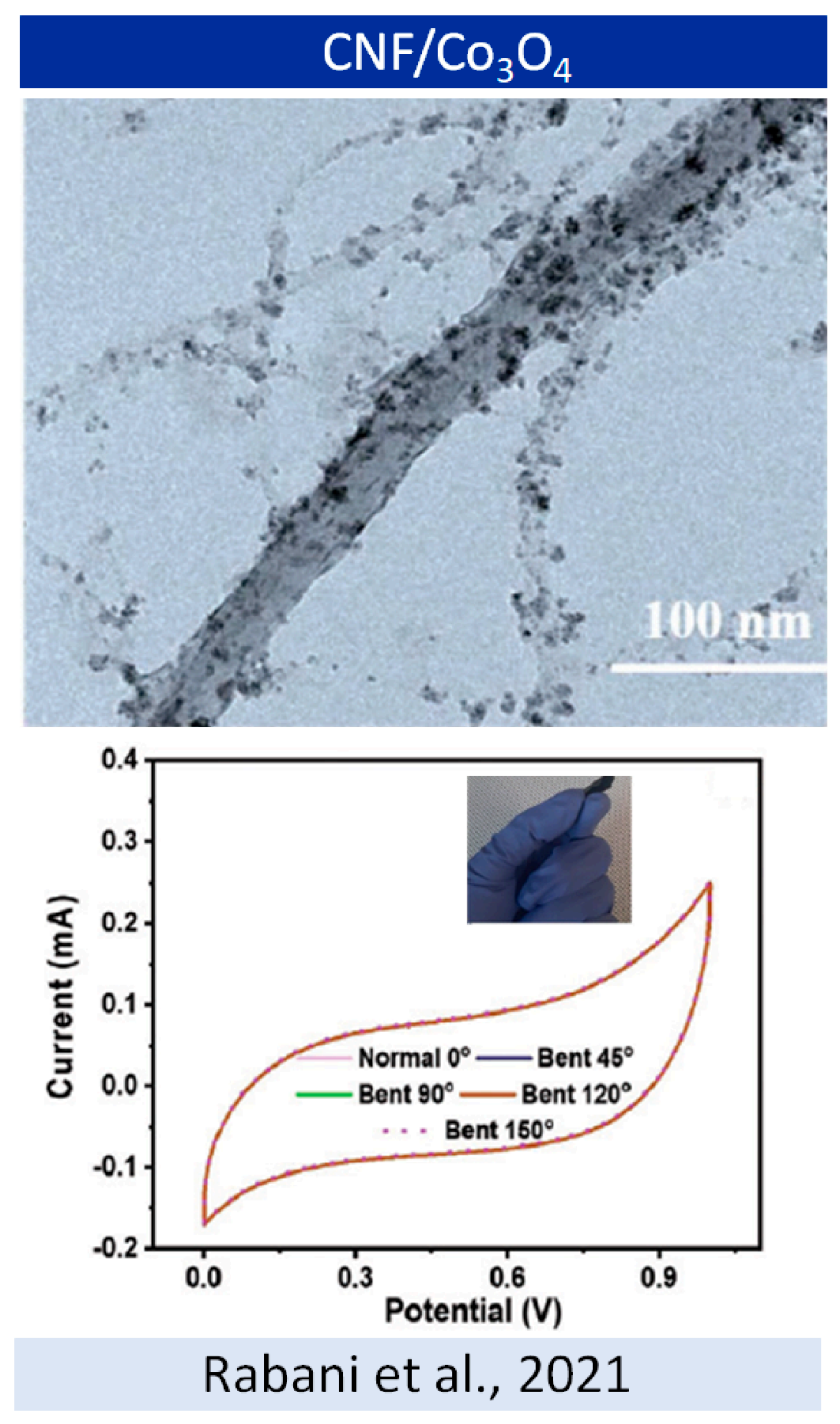
Representative SEM and TEM micrographs and demonstrations of device operation reported for natural polymer–cobalt-based ceramic composite electrodes for flexible energy-storage applications: Co_3_O_4_/CNF electrode, showing Co_3_O_4_ nanoparticles uniformly distributed and attached along the CNF surface (**top**), and CV profiles recorded at 20 mV s^−1^ under normal and bent states at different angles (**bottom**). Reproduced from Refs [61] according to the copyright permissions.

Representative morphological features and a demonstration of device operation reported for selected Co_3_O_4_-based natural polymer composite electrodes are presented in Figure 7. Table 5 summarizes the key electrochemical properties and flexibility-related mechanical performance of the formulations identified by the authors as optimal or best-performing in the studies discussed above.

#### 3.1.5. Zinc- and Titanium-Based Electrodes

The Zn-based series begins with the work of Rafiq et al. [63], who developed flexible composite paper electrodes by combining lignocellulosic (LC) fibers extracted from *Monochoria vaginalis* with ZnO/MnO_2_ and ZnO/TiO_2_ nanostructures. The binary ZnO/MnO_2_ and ZnO/TiO_2_ composites were synthesized through consecutive hydrothermal steps and subsequently incorporated into the lignocellulosic fiber network to produce flexible bulk-paper electrodes.

The LC fibers acted as a binder for the ZnO-based composites, enabling the fabrication of flexible bulk-paper electrodes. In the ZnO/TiO_2_/LC system, electrochemical characterization was performed by cyclic voltammetry using 2.0 M Na_2_SO_4_, confirming the electroactive behaviour of both ZnO/MnO_2_/LC and ZnO/TiO_2_/LC electrodes.

Although the study demonstrated the feasibility of using lignocellulosic fibers to fabricate flexible paper-based electrodes, the electrochemical evaluation remained relatively limited. No specific capacitance, energy density, or power density values were reported. Cycling stability was evaluated only over 100 cycles, with ZnO/MnO_2_/LC retaining approximately 70% of its initial capacitance, whereas ZnO/TiO_2_/LC showed a capacitance loss of 13%, corresponding to approximately 87% retention. Moreover, despite the flexible nature of the composite sheets, no quantitative mechanical characterization or electrochemical testing under bending was reported.

This limits the extent to which the ZnO-based lignocellulosic electrodes can be compared quantitatively with the other systems reviewed here. The different capacitance retention observed for ZnO/MnO_2_/LC and ZnO/TiO_2_/LC suggests that the ceramic composition influences electrochemical stability; however, the absence of quantitative capacitance values and quantitative mechanical characterization, together with the limited evidence linking the structural features to electrochemical performance, prevents the individual contributions of the lignocellulosic scaffold and the ceramic phases from being clearly separated.

Following the ZnO-containing lignocellulosic composite papers described above, Rabani et al. [64] developed a more controlled hybrid architecture based on one-dimensional cellulose nanofibers (1D-CNFs) decorated with uniformly distributed ZnO nanoparticles. The ZnO nanoparticles were synthesized directly in the presence of CNFs through a sol–gel process, with the hydrophilic CNF network promoting their dispersion and limiting particle aggregation. Different formulations were prepared by varying the ZnO precursor concentration. Among the investigated formulations, 1D-CNF@ZnO-3, containing 68 wt% ZnO, exhibited the highest electrochemical performance and was subsequently evaluated in a solid-state symmetric supercapacitor configuration, with the 1D-CNF@ZnO hybrid supported on Ni foam. The device delivered a specific capacitance of 220 F g^−1^ at 1 A g^−1^ and an energy density of 30.2 Wh kg^−1^ at a power density of 1 kW kg^−1^, and retained 88% of its initial capacitance after 8000 cycles. To obtain a fully paper-based flexible device, homogeneous 1D-CNF@ZnO suspensions were subsequently vacuum-filtered to produce self-supporting films, which were carbonized at 250 °C for approximately 30 min under a H_2_/Ar atmosphere prior to device assembly. XRD analysis confirmed the persistence of cellulose after carbonization. The persistence of cellulose after the low-temperature treatment is particularly relevant when interpreting the role of the polymer, as the resulting material should not be regarded simply as a conventional carbonized carbon electrode. Rather, the treatment modifies the electrode structure and electrical response while retaining a cellulose-containing framework.

The resulting carbonized self-supporting films were then assembled into a symmetric solid-state supercapacitor using a PVA–KOH gel electrolyte. Carbonization substantially increased the CV current response and integrated area, indicating an enhanced capacitive response. The optimized flexible device delivered a specific capacitance of 140 F g^−1^ at 1 A g^−1^ and a maximum energy density of 25.2 Wh kg^−1^ at a power density of 1080 W kg^−1^, and retained 92% of its initial capacitance after 8000 charge–discharge cycles. Additionally, the flexibility of the device was also experimentally evaluated: the CV response remained essentially unchanged under 90° bending and showed no significant decay in current response or integrated area after 100 bending cycles.

Compared with the earlier lignocellulosic/ZnO systems, the controlled CNF architecture allows a clearer assessment of the relationship between ZnO loading, nanoparticle dispersion, and electrochemical performance. Nevertheless, the substantial difference between the non-carbonized solid-state device and the carbonized flexible-device performance again demonstrates that electrochemical metrics obtained from different device architectures and processing conditions cannot be assumed to be directly comparable. In addition, the carbonization treatment introduces an important processing variable that modifies the electrical and structural characteristics of the cellulose-based scaffold and should therefore be considered when comparing this system with non-carbonized cellulose electrodes.

Building on their previous work on cellulose nanofiber-based metal oxide electrodes, Rabani et al. [65] extended this strategy by introducing boron nitride nanotubes (BNNTs) into a ZnO/CNF system, aiming to simultaneously enhance the electrochemical and piezoelectric performance of flexible paper-based devices. ZnO nanoparticles were synthesized on the surfaces of both CNFs and BNNTs through a hydrothermal route, producing a BNNT–CNF/ZnO ternary nanostructure in which the one-dimensional CNFs and BNNTs acted as structural junctions that promoted ZnO dispersion and limited nanoparticle aggregation. For comparison, pristine BNNTs, pristine ZnO, BNNT–CNF, and BNNT–ZnO were also investigated. In the three-electrode configuration, the BNNT–CNF/ZnO ternary electrode exhibited the highest electrochemical performance, reaching a specific capacitance of 968 F g^−1^ at 1 A g^−1^, compared with 628 F g^−1^ for BNNT–ZnO, 284 F g^−1^ for BNNT–CNF, 241 F g^−1^ for pristine ZnO, and 122 F g^−1^ for pristine BNNTs. The improved electrochemical response was associated with lower solution and charge-transfer resistances, together with rapid charge transfer at high frequencies and lower ionic diffusion resistance at low frequencies.

This systematic comparison provides stronger evidence for assigning the enhanced electrochemical response to the combined BNNT–CNF/ZnO architecture than is possible in studies based only on an optimized composite. The progressively higher capacitance from pristine BNNT and ZnO to the binary and ternary systems suggests that the conductive and structural networks jointly contribute to the electrochemical response. Importantly, however, the comparison also indicates that the observed enhancement should be interpreted as an architectural effect rather than as a simple consequence of ZnO incorporation, since the performance depends on the simultaneous presence and interaction of CNF, BNNTs, and ZnO.

The ternary material was subsequently evaluated in a two-electrode symmetric configuration using a gel electrolyte. The BNNT–CNF/ZnO device delivered a specific capacitance of 300 F g^−1^ at 1 A g^−1^, retained 96% of its initial capacitance after 5000 cycles at 20 A g^−1^, and achieved an energy density of 37.25 Wh kg^−1^ at a power density of 0.9 kW kg^−1^. For the fabrication of an ultra-thin flexible device, the BNNT–CNF/ZnO suspension was processed into free-standing paper through vacuum-assisted filtration, followed by drying, peeling from the filter membrane, and carbonization at 200 °C for 25 min. Despite this thermal treatment, XRD analysis of the flexible paper after electrochemical cycling still revealed diffraction peaks attributed to CNF, indicating that the cellulose component was not completely removed during processing. Two identical carbonized papers were subsequently employed as the electrodes in a flexible solid-state symmetric supercapacitor. The resulting device achieved a specific capacitance of 94 F g^−1^ at 1 A g^−1^ and retained 97% of its initial capacitance after 5000 cycles. It also delivered an energy density of approximately 30.7 Wh kg^−1^ at a power density of 1350 W kg^−1^. Flexibility was further evaluated by CV measurements at bending angles ranging from 0° to 180°, with the electrochemical response remaining nearly unchanged.

As observed for the previous ZnO/CNF system, the transition from three-electrode measurements to a two-electrode device and subsequently to a fully flexible paper-based device is accompanied by a substantial decrease in specific capacitance. This progressive reduction highlights the influence of device architecture and testing configuration on the reported electrochemical metrics and reinforces the need to distinguish electrode-level performance from practical flexible-device performance. In this case, the transition to the flexible configuration also involves carbonization of the paper-based electrodes, introducing an additional processing variable that may further affect the measured performance.

The work also explored the piezoelectric properties of the flexible papers. The BNNT content was varied from 3 to 12 wt%, with the ternary paper showing an increasing piezoelectric response as the BNNT content increased.

Moving from ZnO-based flexible energy-storage electrodes to TiO_2_-based systems, Wang et al. [66] developed free-standing and mechanically robust film electrodes for sodium-ion batteries using a hierarchical architecture composed of TiO_2_ nanoparticles and carbon nanotube (CNT) networks embedded within sheet-like cellulose. The electrode was assembled from porous TiO_2_–CNT@cellulose nanosheet subunits, in which the abundant functional groups and large surface area of the cellulose promoted the uniform dispersion of TiO_2_ nanoparticles and the interweaving of CNTs, while the subsequent stacking of these subunits through van der Waals interactions and hydrogen bonding produced a compact, self-supporting film. Three TiO_2_–C films containing nominally 65, 75, and 85 wt% TiO_2_ were investigated. Increasing the TiO_2_ content enhanced the active-material loading but progressively reduced the mechanical strength and electrical conductivity. The measured TiO_2_ contents were 62.1, 75.3, and 84.3 wt%, corresponding to tensile strengths of 57 ± 3.6, 46 ± 2.4, and 30 ± 4.7 MPa, respectively. The cellulose-containing architecture was essential for maintaining structural integrity, as a control TiO_2_/CNT film without cellulose exhibited extensive cracking after filtration. The 75 wt% TiO_2_ formulation provided the best overall balance between electrochemical performance and mechanical robustness and was therefore selected for detailed electrochemical evaluation. In a sodium half-cell configuration using sodium foil as the counter electrode, the TiO_2_–C film-75 exhibited initial discharge and charge capacities of 232 and 167 mAh g^−1^, respectively, at 0.5 mA cm^−2^, and retained 207 mAh g^−1^ after 100 cycles, corresponding to 95.8% retention relative to the highest capacity. It also delivered a maximum specific capacity of 227 mAh g^−1^ at 0.15 mA cm^−2^ and maintained 72 mAh g^−1^ at 10 mA cm^−2^. Long-term cycling further demonstrated the stability of the optimized electrode, which retained 81.5% of its maximum capacity after 9000 cycles at 5 mA cm^−2^, with a capacity of approximately 84 mAh g^−1^. The TiO_2_–C film-85 showed higher capacity at low current density but inferior high-rate performance, confirming that the 75 wt% TiO_2_ composition offered a more favourable balance between active-material loading and electronic conductivity. This systematic compositional study provides particularly clear evidence of a structure–property–performance trade-off. Increasing TiO_2_ loading increases the amount of electroactive material but simultaneously decreases tensile strength and electrical conductivity. The 75 wt% formulation therefore represents an optimum balance resulting from competing requirements rather than a simple maximum in active-material content. The control experiment without cellulose further supports the structural role of the polymer, demonstrating that cellulose is essential for maintaining film integrity during processing.

At high mass loading, the TiO_2_–C film-75 also reached an areal capacity of 4.5 mAh cm^−2^, retaining 3.7 mAh cm^−2^ after 50 cycles at an electrode mass of 24.3 mg cm^−2^. The films also exhibited extensive mechanical characterization. The TiO_2_–C electrodes could withstand repeated folding at a bending radius of 0.6 mm, with all formulations surviving more than 3000 folding cycles, while the 65 wt% TiO_2_ film remained intact for up to 11,700 cycles. In contrast, the conventional TiO_2_ electrode failed after only 14 folding cycles.

The mechanical data are particularly informative because they demonstrate that the composition selected for the best overall electrochemical balance is not necessarily the mechanically strongest formulation. Rather, the 75 wt% TiO_2_ electrode achieves a compromise between active-material loading, electronic transport, and mechanical integrity. This reinforces the need to evaluate flexible electrodes using multiple performance dimensions rather than ranking materials according to electrochemical capacity alone.

Following the hierarchical TiO_2_/CNT/cellulose electrodes described above, a simpler TiO_2_–cellulose-based architecture was reported by Chikkatti et al. [67] who incorporated TiO_2_ nanoparticles directly into free-standing cellulose acetate (CA) membranes for supercapacitor applications. The composite membranes were prepared by solution casting from an 8 wt% CA solution, with TiO_2_ loadings of 0.5, 1.0, 1.5, and 2.0 wt%, followed by drying and peeling from the casting substrate. The incorporation of TiO_2_ promoted hydrogen-bonding interactions between the hydroxyl groups at the TiO_2_ surface and the functional groups of the CA matrix, contributing to the integration of the nanoparticles within the polymer membrane. The addition of TiO_2_ also modified the semicrystalline structure of CA and enhanced the mechanical properties up to an optimum loading of 1.0 wt%. At this composition, the membrane exhibited a tensile strength of 39.2 MPa and a Young’s modulus of 8016 MPa, compared with 19.5 MPa and 6882 MPa, respectively, for neat CA. However, the elongation at break decreased substantially to 0.299%, indicating increased brittleness. Further increases in TiO_2_ content resulted in reduced mechanical strength, which was attributed to disruption of the CA interfacial interactions and nanoparticle aggregation. Among the investigated formulations, the 1.0 wt% TiO_2_@CA membrane exhibited the best overall electrochemical performance and was selected for detailed evaluation. Electrochemical measurements were performed in a symmetric two-electrode configuration using 1.0 M H_2_SO_4_ as the electrolyte, with the TiO_2_@CA membranes pressed between nickel-foam layers to ensure electrical contact. The optimized membrane delivered a specific capacitance of 98.15 F g^−1^ at 0.10 A g^−1^, decreasing to 55.38 F g^−1^ at 0.40 A g^−1^. It reached a maximum energy density of 23.03 Wh kg^−1^ at a power density of 0.26 kW kg^−1^, while an energy density of 13 Wh kg^−1^ was maintained at 1.04 kW kg^−1^. The optimized electrode retained 88% of its initial capacitance after 5000 charge–discharge cycles, with a coulombic efficiency close to 100%. Although the membranes were described as flexible and their mechanical properties were quantitatively characterized, no electrochemical evaluation under bending, folding, or other mechanical deformation was reported. Moreover, the optimized composition exhibited very low elongation at break, highlighting a potential limitation for applications requiring repeated or substantial deformation.

The contrast with the TiO_2_–CNT/cellulose system reported by Wang et al. is instructive. While both studies demonstrate that cellulose-containing architectures can accommodate substantial TiO_2_ incorporation, the mechanical response differs markedly: the hierarchical TiO_2_–CNT/cellulose films retained high tensile strength and exceptional folding durability, whereas the TiO_2_/CA membrane exhibited increased tensile strength and stiffness but very low elongation at break. This indicates that “mechanical reinforcement” and “flexibility” should not be treated as equivalent outcomes and are strongly dependent on polymer type, ceramic loading, conductive-network architecture, and interfacial interactions.

Overall, the Zn- and Ti-based studies demonstrate that the role of the natural polymer extends beyond providing flexibility. Depending on the architecture, cellulose or lignocellulosic components act as binders, dispersing scaffolds, structural networks, and mechanically stabilizing phases, while the ceramic loading and conductive-network configuration determine electrochemical activity and charge transport. Across the reviewed systems, increasing ceramic content does not produce a universal improvement: excessive ZnO or TiO_2_ incorporation can promote aggregation and compromise conductivity, charge storage, or mechanical integrity, depending on the electrode architecture. Instead, optimal performance consistently emerges from a balance between electroactive-material loading and the structural and transport functions provided by the polymer-based network. The comparison also highlights the importance of architecture and testing configuration. Several systems exhibit substantially higher capacitance in three-electrode measurements than in assembled flexible devices, demonstrating that electrode-level performance cannot be directly equated with practical device performance.

**Figure 8:**
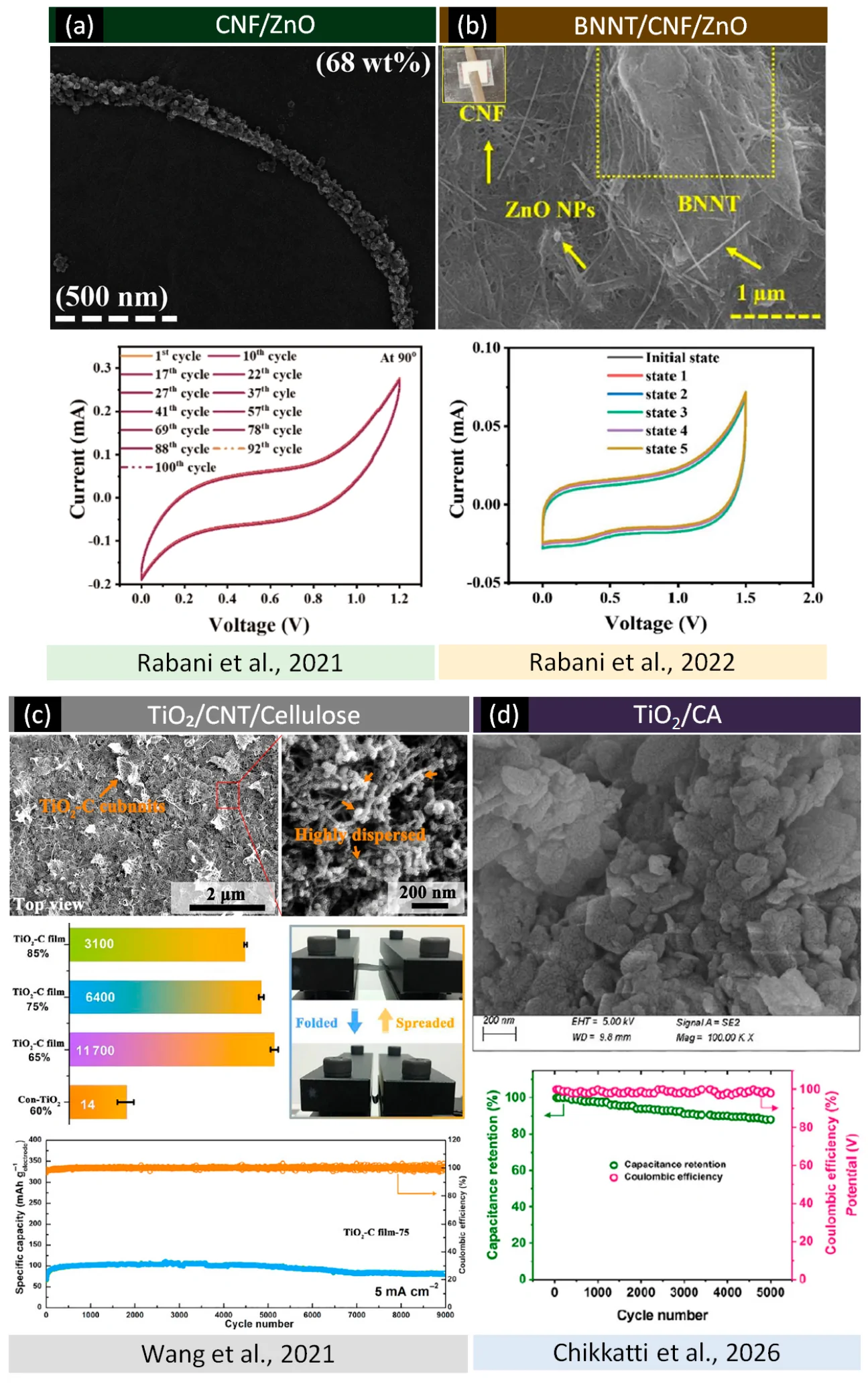
Representative SEM micrographs and demonstrations of device operation reported for natural polymer–zinc- and titanium-based composite electrodes for flexible energy-storage applications. (**a**) CNF/ZnO electrode, showing ZnO nanoparticles uniformly distributed on the surface of the CNFs (**top**) and the CV profiles recorded at a scan rate of 10 mV s^−1^ over 100 bending cycles at a 90° bending angle (**bottom**). (**b**) BNNT–CNF/ZnO electrode, showing the electrode surface after cycling stability, with the inset presenting the flexible device (**top**), and CV curves recorded at different bending angles (0°, 45°, 90°, 120°, 150°, and 180°) to assess its flexibility within a voltage range of 0–1.5 V (**bottom**). (**c**) TiO_2_/CNT/cellulose electrode, showing the homogeneous distribution of TiO_2_ particles within the CNT/cellulose network (**top**), the folding and spreading behaviour of films with different TiO_2_ contents (**middle**), and the long-term cycling performance of TiO_2_–C film-75 over 9000 cycles at 5 mA cm^−2^ (**bottom**). (**d**) TiO_2_/CA electrode, showing the homogeneous distribution of TiO_2_ particles and the interconnected structure of the 1.0 wt% TiO_2_@CA membrane (**top**), and its cycling stability over repeated charge–discharge cycles (**bottom**). Reproduced from Refs [64,65,66,67] according to the copyright permissions or Creative Commons licenses of the original publications.

Representative SEM micrographs and demonstrations of device operation reported for selected natural polymer–zinc- and titanium-based ceramic composite electrodes are presented in Figure 8. Table 6 summarizes the key electrochemical properties and flexibility-related mechanical performance of the formulations identified by the authors as optimal or best-performing in the studies discussed above.

#### 3.1.6. Other Ceramic-Based Electrodes

Beyond the systems discussed above, CuO has also been incorporated into natural polymer-based flexible electrodes. In this context, Peng et al. [68] used bacterial cellulose (BC) as a three-dimensional nanofibrous scaffold for the in situ growth of CuO nanoparticles, followed by the deposition of polypyrrole (PPy) through in situ oxidative polymerization to produce flexible PPy/CuO/BC composite membranes. The porous three-dimensional BC network provided sites for the anchoring and distribution of Cu^2+^ ions, which interacted with the hydroxyl groups of cellulose and subsequently served as active and growth sites for CuO nanoparticles along the BC nanofibers. PPy nanoparticles were then formed predominantly along the BC nanofibers, constructing a continuous nanosheath structure. Four formulations were prepared using Cu(Ac)_2_ precursor concentrations of 0.5, 1, 2, and 5 wt%, designated PPy/CuO/BC-0.5, -1, -2, and -5, respectively. Although PPy/CuO/BC-2 exhibited the highest initial capacitance, reaching 676 F g^−1^ at 0.8 mA cm^−2^, its capacitance decreased sharply during cycling. In contrast, PPy/CuO/BC-1 provided the best overall balance between electrochemical performance and cycling stability and was therefore selected as the representative formulation.

The comparison among the different CuO precursor concentrations indicates that increasing CuO incorporation does not necessarily improve the overall electrode performance. Although PPy/CuO/BC-2 exhibited the highest initial capacitance, its rapid degradation resulted in a less favourable overall performance than the lower-loading formulation. This suggests that the optimum CuO content is governed by a trade-off between the availability of redox-active sites and the structural and interfacial stability of the composite.

The PPy/CuO/BC-1 electrode delivered a specific capacitance of 601 F g^−1^ at 0.8 mA cm^−2^, together with an energy density of 48.2 Wh kg^−1^ at a power density of 85.8 W kg^−1^. After 300 charge–discharge cycles, its capacitance decreased to 385 F g^−1^, corresponding to approximately 64% retention relative to the initial value. The authors therefore acknowledged that the cycling stability remained limited and required further improvement. The relatively low capacitance retention of approximately 64% after only 300 cycles also indicates that high initial capacitance alone is insufficient to establish the suitability of a flexible electrode for long-term operation.

The composite membranes exhibited good qualitative flexibility, with a thickness of approximately 200 μm, and could be bent to 180° and repeatedly folded and released without visible fracture. However, no quantitative mechanical characterization or electrochemical evaluation under mechanical deformation was reported.

Following the PPy/CuO/BC composite described above, Indumathi et al. [69] reported a simpler CuO–chitosan system for supercapacitor applications. CuO nanoparticles were first synthesized by co-precipitation and subsequently incorporated into a chitosan matrix, producing CS–CuO nanocomposites with CS:CuO weight ratios of 1:0.2, 1:0.5, and 1:1. After casting and drying, the composites were subjected to a thermal treatment at 400 °C for 2 h. Among the investigated compositions, increasing the CuO content progressively enhanced the electrochemical response, with the 1:1 CS:CuO formulation exhibiting the largest CV area and the highest specific capacitance. At a scan rate of 20 mV s^−1^, the specific capacitances of the 1:1, 1:0.5, and 1:0.2 formulations were 317, 215.3, and 139.5 F g^−1^, respectively. The optimized CS–CuO electrode delivered a specific capacitance of 325 F g^−1^ at 1 A g^−1^, compared with 115 and 185 F g^−1^ for pristine CS and CuO, respectively. The authors attributed the enhanced performance to the combined contribution of the chitosan matrix and CuO nanoparticles, with the latter providing pseudocapacitive charge-storage behaviour. The electrode also exhibited a capacitance retention above 99% after 10,000 GCD cycles at 1 A g^−1^, together with an energy density of 11.28 Wh kg^−1^ and a power density of 929.57 W kg^−1^. Unlike the previous PPy/CuO/BC system, however, this study did not investigate a free-standing flexible electrode or evaluate its mechanical behaviour. Electrochemical measurements were performed in a three-electrode configuration using a slurry composed of 80 wt% CS–CuO active material, 10 wt% carbon black, and 10 wt% PVDF, coated onto nickel foam. Therefore, despite the use of a natural polymer–ceramic composite as the active material, no flexibility-related performance was demonstrated for the final electrode.

The comparison between the two CuO-based systems further illustrates the importance of the polymer matrix, electrode architecture, and testing configuration. While both studies use CuO as the electroactive ceramic, the BC/PPy architecture provides a free-standing flexible electrode but exhibits limited cycling stability, whereas the CS–CuO system achieves substantially higher cycling retention but relies on a slurry-coated Ni-foam electrode containing PVDF and carbon black. Moreover, the PPy/CuO/BC and CS–CuO systems were evaluated using two-electrode and three-electrode configurations, respectively. Therefore, the superior cycling stability reported for the latter cannot be interpreted independently of the different electrode architecture, conductive/binding components, and testing configuration, and it does not provide equivalent evidence of flexible-device performance.

Expanding the range of ceramic materials incorporated into natural polymer-based electrodes, Figueroa-Gonzalez et al. [70] developed a flexible and predominantly biodegradable supercapacitor based on compacted natural coconut fibers coated with graphene nanoplatelets. Rather than carbonizing the lignocellulosic fibers, the authors used a graphene ink containing pectin to render the coconut-fiber sheets electrically conductive, producing the FG electrodes. MgTiO_3_ nanoparticles, synthesized by a sol–gel method, were then deposited onto one of the graphene-coated coconut-fiber electrodes using a PMMA-containing slurry, yielding the MgTiO_3_-modified FGT electrode. The final flexible solid-state cells were assembled in an asymmetric configuration, combining an FG electrode with an FGT electrode, a rice-paper separator, and either a PVA/H_3_PO_4_ or PVA/OES gel electrolyte. The device was encapsulated with a gelatin/pectin mixture, resulting in a structure in which approximately 95% of the cell components were biodegradable, although the MgTiO_3_ nanoparticles and copper current collectors were not biodegradable. Among the investigated device configurations, FG/OES/FGT, combining the MgTiO_3_-modified electrode with the oral electrolyte solution (OES)-based gel electrolyte, exhibited the highest electrochemical performance. The device delivered a specific capacitance of 807 F g^−1^ at 1 A g^−1^, a specific energy of 161.4 Wh kg^−1^, and a power density of 16.2 W kg^−1^. The incorporation of MgTiO_3_ introduced redox-active charge-storage behaviour and increased both capacitance and specific energy relative to the corresponding MgTiO_3_-free cell. The device also demonstrated flexibility under repeated bending, with FG/OES/FGT retaining 91% of its initial capacitance after 500 bending cycles, representing the highest mechanical–electrochemical stability among the investigated configurations. However, its charge–discharge cycling stability was more limited, with the OES-based cells retaining between 62% and 68% of their initial capacitance after 1000 cycles.

The distinction between mechanical and electrochemical durability is particularly evident in this system. Although the device retained 91% of its capacitance after 500 bending cycles, its capacitance retention after 1000 charge–discharge cycles was only 62–68%. Thus, high electrochemical retention under repeated mechanical deformation does not necessarily imply long-term electrochemical cycling stability. This distinction is important when assessing flexible energy-storage devices, since mechanical robustness and electrochemical durability represent different failure modes and should be evaluated independently.

Continuing the exploration of chitosan-based ceramic composite electrodes, Chikkatti et al. [71] developed freestanding membranes incorporating MoS_2_ nanoparticles for supercapacitor applications. Although MoS_2_ is not a conventional ceramic but rather a transition-metal dichalcogenide, it shares several characteristics with ceramic materials. Chitosan membranes containing 0.5, 1.0, 1.5, and 2.0 wt% MoS_2_ were prepared by dispersing the nanoparticles in a chitosan solution, followed by casting and natural drying. The incorporation of MoS_2_ modified the morphology and mechanical properties of the chitosan membranes. The authors reported changes in the membrane pore structure upon MoS_2_ incorporation, whereas at the highest loading of 2 wt%, significant nanoparticle agglomeration and clustering were observed. Mechanically, the tensile strength and Young’s modulus increased with MoS_2_ content up to 1.5 wt%, while the elongation at break decreased. The 1.5 wt% MoS_2_@CS membrane also exhibited the highest tensile strength and Young’s modulus, reaching 43.3 MPa and 11,162 MPa, respectively, although its elongation at break decreased to 1.05%.

The mechanical results again demonstrate that reinforcement should not be equated with improved flexibility. Increasing MoS_2_ content enhanced tensile strength and stiffness up to 1.5 wt%, but this was accompanied by a reduction in elongation at break. Thus, the 1.5 wt% composition provides enhanced mechanical strength and stiffness, rather than an unequivocal improvement in deformability. For flexible energy-storage applications, both parameters should be considered because a stiffer and stronger membrane may nevertheless be less capable of accommodating large or repeated strains.

Among the investigated formulations, the 1.5 wt% MoS_2_@CS membrane showed the best electrochemical performance. At a scan rate of 30 mV s^−1^, it exhibited the largest CV area among the tested formulations, while GCD measurements yielded a specific capacitance of 345.8 F g^−1^ at 0.2 A g^−1^. The optimized membrane also achieved a maximum energy density of 22.2 Wh kg^−1^ at a power density of 272 W kg^−1^, while a maximum power density of 544 W kg^−1^ was reported at an energy density of 7.2 Wh kg^−1^. The optimized 1.5 wt% MoS_2_@CS membrane further retained 96% of its initial capacitance after 5000 charge–discharge cycles at 0.4 A g^−1^. Although the study described the membranes as freestanding and flexible and quantitatively characterized their tensile properties, no electrochemical evaluation under bending or other mechanical deformation was reported.

Despite the quantitative mechanical characterization, the absence of electrochemical testing under bending prevents direct assessment of whether the improved tensile properties translate into mechanical–electrochemical stability during operation.

Further expanding chitosan-based ceramic hybrid electrodes, Vanitha et al. [72] developed a flexible Ni-doped CeO_2_/chitosan composite in which Ni–CeO_2_ nanoparticles were incorporated into a chitosan matrix. Ni-doped CeO_2_ nanoparticles were first synthesized by co-precipitation and subsequently dispersed in a 1 wt% chitosan solution. The resulting suspension was vacuum-filtered, neutralized with NaOH, washed, and dried to obtain the Ni–CeO_2_@CS hybrid film. Structural characterization confirmed the presence of the semi-amorphous chitosan phase together with the crystalline Ni-doped CeO_2_ nanoparticles, indicating that the biopolymer remained present in the final hybrid material. For electrode fabrication, the Ni–CeO_2_@CS composite was mixed with carbon black and PVDF in an 80:10:10 weight ratio and coated onto nickel foam. The hybrid electrode exhibited the highest electrochemical performance among pristine CeO_2_, Ni–CeO_2_, and Ni–CeO_2_@CS, delivering a specific capacitance of 780 F g^−1^ at 1 A g^−1^ and retaining 650 F g^−1^ at 10 A g^−1^. It also retained approximately 90% of its initial capacitance after 5000 charge–discharge cycles. The Ni–CeO_2_@CS electrode was subsequently assembled as the positive electrode in a flexible asymmetric solid-state supercapacitor using activated carbon as the negative electrode and a PVA/KOH gel electrolyte. The complete device delivered a specific capacitance of 190 F g^−1^ at 1 A g^−1^, while Ragone analysis showed a maximum energy density of approximately 65 Wh kg^−1^ at 0.8 kW kg^−1^. The flexibility of the assembled device was quantitatively evaluated under bending angles of 45°, 90°, and 180°. Capacitance retention remained at 98%, 96%, and 94%, respectively, demonstrating limited electrochemical degradation even under severe deformation. The asymmetric device also retained approximately 90% of its initial capacitance after 5000 charge–discharge cycles at 5 A g^−1^.

The inclusion of pristine CeO_2_ and Ni–CeO_2_ controls provides a more meaningful basis for evaluating the contribution of the chitosan-containing architecture than is available in studies reporting only the optimized composite. The higher capacitance of Ni–CeO_2_@CS indicates that incorporation of Ni–CeO_2_ into the chitosan matrix is associated with an enhanced electrochemical response; however, because carbon black and PVDF are also present in the final electrode, the specific contribution of the chitosan–ceramic interaction cannot be completely isolated from the effects of the conductive additive and binder.

Finally, Aswathy et al. [73] reported a flexible three-dimensional scaffold-like electrode based on a cellulose acetate (CA)/chitosan (CS) blend incorporating a hybrid rGO/NiO/Fe_3_O_4_ nanocomposite and polyaniline (PANI). The CA/CS matrix was prepared by blending CA and CS in a 90:10 mass ratio, followed by phase inversion to generate a porous three-dimensional scaffold-like membrane. The rGO/NiO/Fe_3_O_4_ hybrid nanocomposite, referred to as rNF, was incorporated into the CA/CS blend prior to the in situ polymerization of aniline, yielding the CA/CS/PANI/rNF (CCPRNF) composite membrane. Different rNF loadings of 1, 5, and 15 wt% were investigated, with the 15 wt% CCPRNF formulation exhibiting the highest electrochemical performance. Further increases in rNF loading were expected to improve electrochemical performance but were reported to adversely affect membrane flexibility. This behaviour reinforces the recurring trade-off between electrochemical activity and mechanical compliance observed across the reviewed electrode systems. Increasing the electroactive rNF fraction improves charge-storage performance, while higher rNF loading beyond 15 wt% was reported to adversely affect membrane flexibility. Thus, the optimum formulation is determined by competing electrochemical and mechanical requirements rather than by simply maximizing the rNF content.

The optimized electrode exhibited an areal capacitance of 16.61 mF cm^−2^ at 5 mV s^−1^, decreasing to 2.87 mF cm^−2^ at 300 mV s^−1^, together with an electrical conductivity of 7.1 × 10^−2^ S cm^−1^. The 15 wt% CCPRNF electrodes were subsequently assembled into a symmetric all-solid-state supercapacitor using nickel foam current collectors and a PVA–NaNO_2_ gel electrolyte. The device achieved an areal capacitance of 5.5 mF cm^−2^ at 20 mV s^−1^, retained 71.26% of its initial capacitance after 1000 cycles, and delivered an areal energy density of 0.35 mWh cm^−2^ at an areal power density of 15 mW cm^−2^. Flexibility was further assessed electrochemically by recording CV curves while the device was bent to 90° and 120°. Only slight changes in the CV response were observed, indicating that the device maintained its electrochemical behaviour under these deformation conditions. However, no quantitative capacitance-retention values or repeated bending-cycle stability were reported. This absence of quantitative capacitance retention during bending or repeated deformation limits the strength of the evidence for operational flexibility, despite the slight changes observed in CV profiles at selected bending angles.

Overall, these additional ceramic-based systems reinforce the trends identified for the transition-metal oxide electrodes discussed above: electrochemical performance depends not simply on ceramic loading, but on its integration with the natural polymer matrix and conductive network. The studies further highlight that the optimum composition is system-dependent, with electrochemical activity needing to be balanced against structural integrity, conductivity, and stability.

Table 7 summarizes the key electrochemical properties and flexibility-related mechanical performance of the formulations identified by the authors as optimal or best-performing in the studies discussed above.

Collectively, these studies demonstrate that natural polymers, particularly cellulose and chitosan, provide versatile and adaptable platforms for the development of ceramic-based composite electrodes for flexible energy-storage devices. Among the applications investigated, flexible supercapacitors clearly dominate, with ceramic nanofillers contributing to electrochemical activity, ion-accessible surface area, structural stability, and, in some cases, mechanical reinforcement. However, the reported performance depends strongly on the combined effects of the polymer matrix, ceramic composition and loading, conductive additives or additional polymers, electrode architecture, and device configuration, making direct comparison between individual systems challenging. Beyond conventional energy-storage performance, several studies also highlight additional functionalities, including self-healing behaviour and sensing capabilities, illustrating the potential of these composites for the development of multifunctional flexible and wearable devices.

Nevertheless, despite the widespread use of the term “flexible”, the mechanical performance of many electrode systems remains insufficiently evaluated. While some studies report capacitance retention under repeated bending, compression, or folding, others assess flexibility through electrochemical measurements performed at selected deformation states or angles. In contrast, several studies rely solely on photographic demonstrations, and some do not provide any flexibility-related characterization despite targeting flexible energy-storage applications. Similarly, although sustainability is frequently presented as a key advantage of natural polymer-based electrodes, its actual environmental performance remains largely unexplored, with only one of the studies reviewed here explicitly assessing this aspect.

### 3.2. Natural Polymer–Ceramic Composite Polymer Electrolytes

Although considerable progress has been achieved in the development of electrode materials for flexible energy-storage devices, the electrolyte remains a critical component because it must simultaneously ensure efficient ion transport, electrochemical stability, and mechanical integrity under repeated deformation. Conventional liquid electrolytes generally provide high ionic conductivity but are unsuitable for flexible and wearable devices due to leakage risks, the need for rigid encapsulation, and, depending on the solvent, safety concerns. Consequently, increasing attention has been devoted to both quasi-solid-state electrolytes, such as gel polymer electrolytes (including ionogels, hydrogels, and organogels), and all-solid-state electrolyte membranes, which provide the mechanical robustness required for flexible devices. While gel electrolytes offer improved ionic transport owing to their liquid phase, solid-state membranes provide enhanced dimensional stability and safety. However, each approach presents intrinsic limitations, including insufficient mechanical strength or ionic conductivity in polymer-based systems and the brittleness of ceramic electrolytes. To address these challenges, polymer–ceramic composite electrolytes have emerged as an effective strategy by combining the flexibility and processability of polymer matrices with the mechanical reinforcement and electrochemical benefits provided by inorganic fillers. Natural polymer–ceramic composites are particularly attractive owing to their renewability, low environmental impact, abundance, and versatile chemical functionality [85,86,87,88,89]. However, natural polymers are rarely employed as the sole polymeric matrix. Instead, they are often blended with synthetic polymers to improve mechanical and electrochemical performance, partially offsetting the sustainability advantages associated with the use of renewable polymer matrices.

The following studies illustrate the limited but emerging body of literature on natural polymer–ceramic composite electrolytes for flexible batteries and supercapacitors, highlighting how natural polymers have been combined with ceramic fillers and, in many cases, complementary synthetic polymeric components.

An early example of a natural polymer–ceramic composite electrolyte for flexible energy-storage applications was reported by Guo et al. [74], who developed a cellulose-based bionanocomposite ionogel through in situ chemical crosslinking of cellulose dissolved in 1-ethyl-3-methylimidazolium acetate ([EMIM][OAc]) with bisphenol A epoxy resin (E44) via ring-opening reactions initiated by cerium ammonium nitrate, while HNTs were incorporated as ceramic nanofillers prior to gelation. This fabrication strategy produced chemically crosslinked liquid-crystalline ionogels in which the incorporation of HNTs generated a porous microstructure with aligned nanotube-rich channel walls, providing ion-transport pathways and resulting in enhanced ionic conductivity, mechanical properties, and thermal stability. The optimized formulation (5% cellulose and 15 wt% HNTs) exhibited a room-temperature ionic conductivity of 1.6 mS cm^−1^, a tensile strength of 3.2 MPa, and remained mechanically stable up to 200 °C. A subsequent shear-alignment step further increased the ionic conductivity to 2.5 mS cm^−1^ by orienting the HNTs and promoting continuous ion-transport pathways. The assembled supercapacitor exhibited only 5.7% capacitance loss after 5000 charge–discharge cycles, demonstrating excellent cycling stability. Although the authors highlighted the potential of these ionogels for flexible energy-storage devices and demonstrated electrochemical performance under different bending angles, the flexibility of the electrolyte itself was not quantitatively evaluated through dedicated bending or cyclic flexural tests.

A different strategy for flexible solid-state electrolytes was subsequently proposed by Cai et al. [75], who developed an ultrathin flexible ceramic-based composite electrolyte for lithium metal batteries by combining a lithium aluminum germanium phosphate (Li_1.5_Al_0.5_Ge_1.5_(PO_4_)_3_, LAGP) ceramic with a cellulose mesh. A homogeneous suspension of LAGP and poly(vinylidene fluoride) (PVDF) was first deposited onto the cellulose mesh by solution casting, producing a thin ceramic membrane in which the cellulose acted as a permanent flexible scaffold and PVDF served as a binder. Subsequently, a gel polymer electrolyte composed of poly(ethylene glycol) diacrylate (PEGDA), fluoroethylene carbonate (FEC), and lithium bis(trifluoromethanesulfonyl)imide (LiTFSI) was introduced into the membrane through in situ thermal polymerization, generating a ceramic–gel composite electrolyte with improved electrode/electrolyte interfacial contact. According to the authors, this simple and energy-efficient fabrication route is compatible with large-scale manufacturing and enabled the preparation of an ultrathin (~23 μm) electrolyte with excellent flexibility. The optimized composite electrolyte exhibited an ionic conductivity of 0.112 mS cm^−1^, an ionic conductance of 86.05 mS, a Li^+^ transference number of 0.54, and an electrochemical stability window of 4.7 V. The high LAGP loading, together with the FEC-containing gel interlayer, promoted uniform Li^+^ transport, suppressed lithium dendrite growth, and enabled stable lithium plating/stripping for 300 h in symmetric Li/Li cells. In lithium iron phosphate (LiFePO_4_, LFP)/Li full cells, the composite electrolyte delivered an initial discharge capacity of 140.2 mAh g^−1^ and maintained 151.5 mAh g^−1^ after 180 cycles at 0.5 C with nearly 100% coulombic efficiency. Moreover, pouch cells remained operational under bending, folding and after being cut, suggesting the applicability of the cellulose-supported composite electrolyte for flexible lithium metal batteries, although no quantitative flexibility tests were reported.

Building on their previous cellulose mesh-supported composite electrolyte, Cai et al. [76] further developed this strategy by incorporating tantalum-doped lithium lanthanum zirconate (Li_6.4_La_3_Zr_1.4_Ta_0.6_O_12_, LLZTO) nanoparticles into a poly(ethoxylated trimethylolpropane triacrylate) (poly(ETPTA))-based gel polymer electrolyte reinforced with a cellulose mesh. The composite electrolyte was fabricated through an environmentally friendly in situ thermal polymerization process, in which the ultrathin cellulose mesh acted as a permanent supporting skeleton, yielding a ~40 μm thick electrolyte with a homogeneous distribution of LLZTO nanoparticles throughout the polymer matrix. The authors also reported that thermal treatment transformed the fluid precursor slurry into a flexible solid-state membrane. However, no quantitative evaluation of flexibility or bending durability was performed. The optimized composite electrolyte exhibited an ionic conductivity of 0.652 mS cm^−1^, a Li^+^ transference number of 0.71, and an electrochemical stability window of 4.82 V. According to the authors, the incorporation of LLZTO nanoparticles, together with the poly(ETPTA)-based gel polymer electrolyte, promoted rapid Li^+^ transport, improved interfacial stability, and effectively suppressed lithium dendrite growth, enabling stable Li plating/stripping for 500 h in symmetric cells. In LFP/Li full cells, the electrolyte delivered a capacity retention of 91.6% after 200 cycles at 0.5 C.

In contrast to the cellulose-based systems described above, Sheng et al. [77] developed a sustainable nanocomposite organic ionogel electrolyte for flexible supercapacitors using chitosan reinforced with varying contents of nano-silica (nano-SiO_2_). The electrolyte was prepared through a simple one-pot approach in which nano-SiO_2_ was first dispersed in an organic electrolyte solution (OES) composed of 1-ethyl-3-methylimidazolium acetate ([EMIM][OAc]) and the biomass-derived solvent γ-valerolactone (GVL), followed by dissolution of chitosan at 90 °C. The homogeneous suspension was subsequently cast and exposed to a controlled-humidity environment, inducing a solution-to-gel transition that produced a free-standing and flexible ionogel. The authors proposed that the resulting three-dimensional network is formed by physically cross-linked chitosan chains through hydrogen bonding and chain entanglement, together with hydrogen-bonding interactions between chitosan, nano-SiO_2_ particles, and the organic electrolyte solution (OES), simultaneously providing mechanical reinforcement and continuous ion-transport pathways. The optimized formulation, containing 2 wt% nano-SiO_2_, exhibited an ionic conductivity of 24.3 mS cm^−1^ at 25 °C, increasing to 60.3 mS cm^−1^ at 80 °C while maintaining 2.3 mS cm^−1^ at −25 °C. The incorporation of nano-SiO_2_ also increased the compressive stress from 0.67 MPa for the SiO_2_-free ionogel to 1.52 MPa, while maintaining high deformability. SEM observations of the freeze-dried cryogel, together with elemental mapping, confirmed a homogeneous distribution of nano-SiO_2_ within a highly porous honeycomb-like structure, favourable for electrolyte retention and ion transport. Flexible symmetric supercapacitors assembled with the optimized ionogel delivered a specific capacitance of 82.4 F g^−1^ at 0.5 A g^−1^, maintained stable electrochemical performance over a wide operating temperature range (−25 to 80 °C), and exhibited almost no capacitance decay after 10,000 cycles at 25 °C. Flexibility was demonstrated through repeated twisting and bending of the ionogel, while nearly overlapping cyclic voltammetry curves recorded at bending angles ranging from 0° to 180° confirmed that mechanical deformation had a negligible effect on the electrochemical response, highlighting the potential of this nanocomposite ionogel for flexible energy-storage applications.

More recently, Huo et al. [78] reported a cellulose-based dual-network gel electrolyte (PANa–SiO_2_–CMC) for flexible zinc–air batteries by incorporating carboxymethyl cellulose (CMC) and nano-SiO_2_ into a sodium polyacrylate (PANa) network. The PANa–SiO_2_–CMC gel combines a covalently cross-linked rigid network with an ionically cross-linked flexible network, while interactions between CMC and PANa promote a denser three-dimensional structure. The incorporation of CMC substantially modifies the gel morphology, transforming the lamellar structure of PANa into an interconnected three-dimensional network with increased channel density, which improves electrolyte penetration.

The optimized PANa–SiO_2_–CMC electrolyte exhibited a tensile strength of 3.62 MPa and an elongation of 1865.01%, compared with 2.26 MPa and 1119.34% for PANa alone. Its ionic conductivity reached 276.51 mS cm^−1^, higher than those of PANa (231.09 mS cm^−1^) and PANa–SiO_2_ (247.25 mS cm^−1^). The enhanced ionic transport was attributed to SiO_2_ effectively reducing the aggregation of PANa chains, thereby improving liquid absorption within the hydrogel network and opening up blocked channels, enabling ions to migrate more easily. Flexible zinc–air batteries assembled with the PANa–SiO_2_–CMC electrolyte delivered a capacity of 760.61 mAh g^−1^ and maintained stable voltage output over 160 h. The battery also retained stable electrochemical operation under bending angles from 0° to 180°, with no apparent performance deterioration after repeated bending. The authors further demonstrated practical operation by powering an LED and by connecting three cells in series to obtain an open-circuit voltage of 3.70 V.

Despite the promising mechanical and ionic properties of the PANa–SiO_2_–CMC electrolyte, several aspects relevant to its long-term applicability remain insufficiently characterized. In particular, the study does not report a quantitative thermal-stability assessment or an electrochemical stability window for the gel electrolyte. Although the assembled zinc–air battery exhibited stable operation over 160 h, no quantitative capacity-retention value was provided, limiting direct assessment of its electrochemical durability. Furthermore, the reported 1.4 V value corresponds to the open-circuit voltage of the assembled zinc–air battery rather than to the electrochemical stability window of the electrolyte and should therefore not be interpreted as such. These limitations should be considered when assessing the long-term stability and practical operating range of the proposed cellulose-containing gel electrolyte.

**Figure 9:**
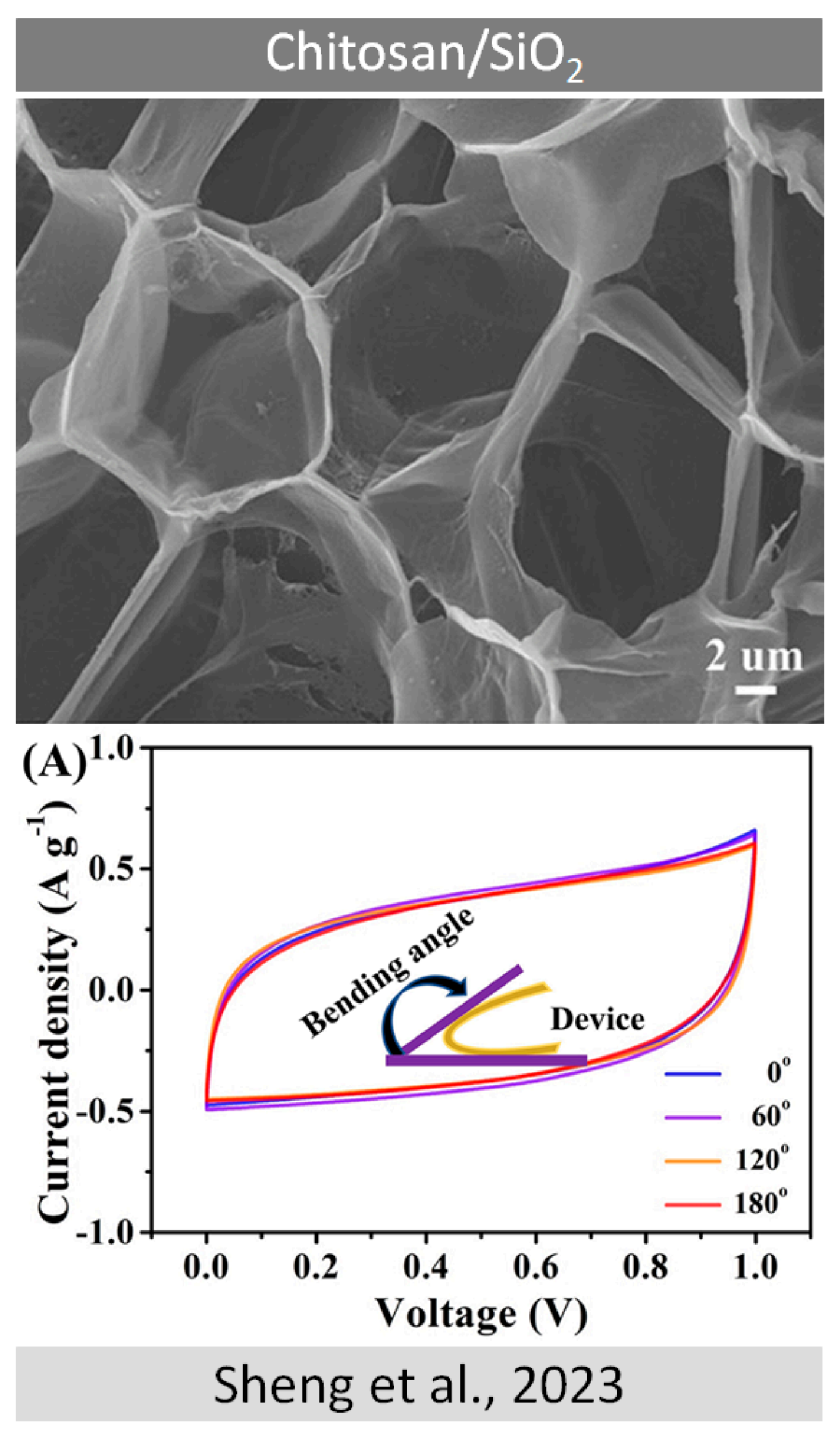
Representative SEM micrograph and experimental demonstrations reported for natural polymer–ceramic composite electrolyte for flexible energy-storage applications: chitosan/SiO_2_, showing the porous honeycomb-like microstructure (**top**) and cyclic voltammetry curves recorded at different bending angles (**bottom**). Reproduced from Ref. [77] according to the copyright permissions.

A representative SEM image and demonstration of flexibility reported for a selected natural polymer–ceramic composite electrolyte are presented in Figure 9.

Additionally, for ease of comparison, the formulations identified by the authors as the best-performing in the studies discussed above, together with their key physicochemical, mechanical, and electrochemical properties, are summarized in Table 8.

Collectively, these studies demonstrate that natural polymers, particularly cellulose and chitosan, provide versatile platforms for the development of flexible composite electrolytes and ionogels for energy-storage devices. The incorporation of ceramic nanofillers not only enhances mechanical robustness but also facilitates ion transport by creating continuous conduction pathways and improving electrolyte/electrode interfacial stability. Overall, the reported systems achieved ionic conductivities, electrochemical stability, and cycling performance suitable for their respective energy-storage applications, although the absolute values varied considerably depending on the polymer matrix, ceramic filler, and device configuration.

Nevertheless, despite their intended application in flexible energy-storage devices, the assessment of mechanical flexibility remains largely qualitative. In most cases, flexibility was inferred from photographic demonstrations or electrochemical measurements under deformation, whereas standardized quantitative evaluations, such as repeated bending, cyclic flexural fatigue, or durability tests, were rarely performed. Future studies should therefore combine electrochemical characterization with systematic mechanical testing to establish reliable structure–property relationships and facilitate the practical implementation of flexible polymer–ceramic electrolytes.

### 3.3. Natural Polymer–Ceramic Composite Separators

Regardless of whether they are employed in batteries or supercapacitors, separators play a fundamental role in ensuring the safe and efficient operation of electrochemical energy storage devices. They act as physical barriers that prevent direct electrical contact between the electrodes, thereby avoiding internal short circuits, while simultaneously providing ion transport pathways through the electrolyte. Consequently, the structure and properties of separators have a profound influence on the electrochemical performance, safety, and cycling stability of both batteries and supercapacitors. An ideal separator should combine high porosity, excellent electrolyte wettability and permeability, superior thermal stability, high mechanical strength and flexibility, compatibility with industrial manufacturing and practical use, and outstanding chemical and electrochemical stability [79,90,91,92].

Commercial batteries predominantly employ microporous polyolefin separators based on polyethylene (PE) and polypropylene (PP), owing to their low cost, excellent chemical resistance, and satisfactory mechanical properties. However, these materials exhibit poor electrolyte wettability because of their hydrophobic nature and possess relatively low thermal stability, with melting temperatures of approximately 130 °C for PE and 165 °C for PP, which may compromise battery safety under abusive operating conditions. In contrast, commercial supercapacitors commonly employ either polyolefin membranes or nonwoven polymer separators. Although nonwoven separators generally exhibit higher porosity and improved electrolyte uptake, they often suffer from inferior mechanical strength and structural reliability owing to their fabrication process. Consequently, both conventional separator systems present intrinsic limitations that hinder their application in next-generation flexible and wearable energy storage devices [92,93].

These limitations have stimulated the search for sustainable alternatives capable of simultaneously improving electrochemical performance and reducing the environmental footprint of energy storage devices. Within the framework of the bioeconomy and circular economy, renewable resources have attracted considerable attention for the development of bio-based polymeric membranes. Derived from plant, animal, or marine biomass, these natural polymers offer advantages such as renewability, biodegradability, abundant availability, and excellent electrolyte affinity, making them promising candidates for next-generation separator materials. Consequently, separators based on biopolymers and other water-processable polymers are increasingly being investigated as environmentally friendly alternatives to conventional petroleum-derived membranes [94,95].

Among natural polymers, cellulose has emerged as the most extensively investigated separator matrix owing to its abundance, low cost, renewability, and excellent physicochemical properties. Cellulose and its derivatives form highly porous fibrous membranes that exhibit superior electrolyte uptake, low interfacial resistance, and excellent dimensional stability at elevated temperatures. In addition to cellulose, other natural polymers, including chitosan, starch, alginate, gelatin, lignin, silk fibroin, and agar, have also demonstrated considerable potential as separator materials because of their intrinsic hydrophilicity, favourable mechanical properties, and good thermal and electrochemical stability. Furthermore, these biopolymers can be readily processed into membranes using scalable and cost-effective techniques, such as electrospinning and solution casting. These biopolymers also enable a wide range of chemical modifications and crosslinking strategies, allowing the surface chemistry, electrolyte affinity, ionic conductivity, and mechanical performance to be tailored for specific energy storage applications [90,94,96].

To further enhance the performance of natural polymer separators, the incorporation of ceramic nanofillers has attracted particular attention owing to their ability to simultaneously improve the thermal stability, mechanical robustness, electrolyte wettability, and safety of natural polymer separators, while maintaining efficient ion transport. The following studies illustrate the evolution of ceramic-reinforced natural polymer separators for flexible batteries and supercapacitors.

One of the earliest examples of ceramic-reinforced natural polymer separators was reported by Li et al. [79], who developed a flexible composite separator by integrating hydroxyapatite (HAp) nanowire networks with cellulose fibers (CFs) through a self-assembly process. The resulting hierarchically cross-linked membrane combined the intrinsic flexibility and high electrolyte affinity of cellulose with the excellent thermal stability and fire resistance of HAp. Compared with commercial polypropylene separators, the HAp/CF membrane exhibited substantially higher electrolyte uptake (253%) and ionic conductivity (3.07 mS cm^−1^), while maintaining structural integrity up to 200 °C and excellent flame resistance. Consequently, lithium-ion batteries assembled with the composite separator delivered improved cycling stability and rate capability, demonstrating the effectiveness of ceramic nanofillers in enhancing both safety and electrochemical performance. Although tensile properties were quantitatively evaluated, flexibility was assessed primarily through qualitative demonstrations (rolling, twisting, folding, and scrunching), without standardized cyclic bending or flexural tests.

Cellulose acetate has also been explored as a separator matrix in combination with TiO_2_ nanoparticles. Yuan et al. [80] fabricated electrospun cellulose acetate/polyvinyl alcohol/titanium dioxide (CA/PVA/TiO_2_) composite separators for flexible zinc–air batteries and subsequently reinforced the membrane through glutaraldehyde (GA) crosslinking to reduce the number of hydroxyl groups in CA and PVA, thereby decreasing the hydrophilicity and improving the mechanical properties of the separator. The incorporation of an optimal TiO_2_ content (2 wt%) promoted the formation of a uniform three-dimensional nanofibrous network with enhanced porosity, reduced contact angle, improved electrolyte uptake, and significantly higher ionic conductivity than the pristine CA/PVA separator, although the mechanical properties were still not satisfactory. To overcome this limitation, the CA/PVA/TiO_2_ separator was subsequently crosslinked with GA, which substantially improved the mechanical properties without compromising electrolyte transport, yielding a separator with a porosity of 68.2%, an electrolyte uptake of 285.3%, an ionic conductivity of 10.36 mS cm^−1^, and excellent tensile properties. As a result, the assembled flexible zinc–air batteries exhibited improved electrochemical performance relative to the pristine CA/PVA separator. While TiO_2_ incorporation was primarily responsible for the marked enhancement in electrolyte transport and battery performance, subsequent GA crosslinking further improved the mechanical properties, provided a modest additional increase in ionic conductivity, and more effectively inhibited the growth of zinc dendrites. Despite the material being presented as a flexible separator for wearable zinc–air batteries, the study did not include qualitative demonstrations (e.g., photographs of bending or folding) or quantitative flexibility tests. Instead, the mechanical characterization was limited to tensile measurements, from which flexibility was indirectly inferred.

Cellulosic paper has also attracted considerable attention as a separator matrix owing to its tunable porosity, excellent electrolyte compatibility, safety, scalability, cost-effectiveness, and sustainability. Different ceramic nanofillers have subsequently been incorporated to tailor its physicochemical and electrochemical properties, including SiO_2_, ZrO_2_, Al_2_O_3_ and BaTiO_3_.

Das et al. [81] developed a cellulose paper-based separator by functionalizing a commercial paper substrate with a chitosan/poly(vinyl alcohol) (PVA)/SiO_2_ composite using a scalable dip-coating process. Four different SiO_2_ loadings were investigated to evaluate the influence of the ceramic content on separator performance. The resulting cellulose–ceramic composite separators exhibited excellent thermal stability (>200 °C) without dimensional shrinkage, outstanding electrolyte wettability (147%) with a zero-contact angle, rapid electrolyte saturation, and satisfactory tensile strength (up to 38.31 MPa). Compared with untreated paper, the ceramic–polymer coating significantly improved the mechanical properties, although the tensile strength remained lower than that of the commercial polypropylene (PP) separator. Electrochemical evaluation in CR2032 coin cells demonstrated performance comparable to that of commercial PP separators over a current density range of 0.05–0.4 mA cm^−2^, with excellent coulombic efficiency (>96%) and good capacity retention during cycling. Furthermore, the separator was compatible with several commercial electrode materials (MCMB, LiCoO_2_ and LiFePO_4_) and exhibited excellent flame-retardant behaviour, highlighting its potential as a sustainable and safer alternative for lithium-ion battery separators. Despite being presented as a flexible paper separator, the study did not include a dedicated flexibility assessment. Although tensile stress–strain measurements were performed to evaluate the mechanical properties, no qualitative demonstrations of flexibility or quantitative bending, folding, or flexural tests were reported.

Extending their previous work on cellulose paper separators, the same research group, Das et al. [82], developed a multilayer cellulose paper separator for lithium-ion batteries and supercapacitors through sequential wet coating of commercial cellulose paper with poly(vinylidene fluoride) (PVDF), followed by ceramic impregnation using a styrene–butadiene rubber (SBR)/BaTiO_3_ composite and a final SBR lamination layer. The fabricated separators exhibited high electrolyte uptake (216–270%), rapid electrolyte saturation, improved mechanical strength (up to 50.15 MPa in the machine direction and 28.21 MPa in the transverse direction), and no dimensional shrinkage up to 200 °C. Calendar-aging tests demonstrated good chemical stability in the organic electrolyte, with no significant morphological degradation or evidence of ceramic particle leaching after eight weeks of storage at room temperature. In a full-cell configuration (MCMB/separator/LiFePO_4_), the composite separator exhibited electrochemical performance comparable to that of a commercial PP/PE separator in terms of specific capacity, rate capability, coulombic efficiency (>96%), and cycling stability over 300 cycles. The separator was also successfully applied in supercapacitors, demonstrating the versatility of the proposed multilayer architecture across different energy storage devices. Although the authors describe the separator as sustainable, economical, and flexible, the incorporation of PVDF (a fluorinated synthetic polymer) partially offsets the sustainability claim. Moreover, despite referring to the separator as flexible, the study evaluated only tensile properties, without performing dedicated flexibility tests such as bending, folding, or cyclic deformation experiments.

Building on the previously developed multilayer paper separator, Sapra et al. (2025) [83] refined the fabrication strategy by reducing the SBR content during both the ceramic impregnation and lamination steps while maintaining the same PVDF/BaTiO_3_ trilayer architecture for application in sodium-ion batteries.

The PVDF coating reduced pore size and improved surface uniformity, while the ferroelectric BaTiO_3_ nanoparticles dispersed within the SBR matrix interacted with the polymer hosts through Lewis acid–base interactions, promoting Na^+^ transport. The resulting membranes exhibited a porous fibrous network with well-dispersed BaTiO_3_ nanoparticles embedded within the polymer-coated cellulose fibers, electrolyte uptake of 152–158%, thermal stability up to 200 °C, and ionic conductivity as high as 2.7 × 10^−4^ S cm^−1^. Among the investigated formulations, the separator laminated with 0.75 wt/v% SBR exhibited the best electrochemical performance, delivering an initial discharge capacity of 114 mAh g^−1^, nearly 100% coulombic efficiency for up to 200 cycles, and 62% capacity retention after 240 cycles, comparable to that of a commercial glass-fiber separator. The authors also demonstrated the separator’s flexibility through bending and folding experiments, complemented by SEM analysis of the folded region, which revealed no significant cracks or disruption of the fibrous network after deformation.

To elucidate the influence of ceramic composition on separator performance, Das et al. (2024) [84] employed the same multilayer cellulose paper architecture to compare Al_2_O_3_-, BaTiO_3_-, and ZrO_2_-reinforced separators for lithium-ion batteries.

The fabrication involved sequential PVDF sizing of the cellulose paper, followed by dip coating with SBR-based ceramic suspensions containing the respective ceramic nanofillers. The incorporation of ceramic nanoparticles significantly enhanced electrolyte uptake, thermal stability, flame retardancy, and electrochemical performance compared with the unmodified paper separator. Although all three ceramic-reinforced separators exhibited excellent cycling stability, rate capability, and coulombic efficiency comparable to those of commercial polyolefin separators, the ZrO_2_-based separator demonstrated the best overall performance owing to its higher Li^+^ transference number, lower charge-transfer resistance, and improved interfacial compatibility with the electrodes. The study also highlighted that the surface charge and deposition behavior of the ceramic nanofillers strongly influenced their interaction with the cellulose matrix and, consequently, the physicochemical and electrochemical performance of the separators.

The flexibility of the developed separators was qualitatively demonstrated through rolling, bending, and twisting experiments. Nevertheless, no quantitative evaluation of their mechanical flexibility or durability under repeated deformation was reported. Furthermore, although the cellulose paper substrate contributes to improving sustainability, the use of a PVDF sizing layer partially offsets this advantage, making the sustainability claims less compelling.

**Figure 10:**
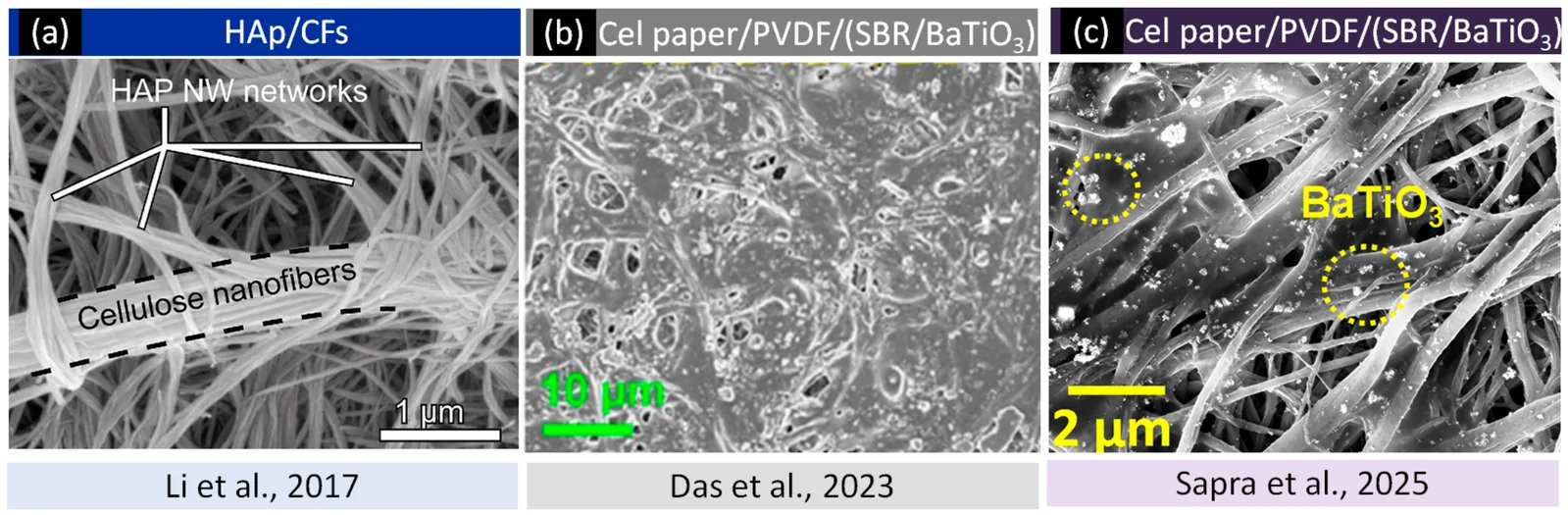
Representative SEM micrographs of ceramic-reinforced natural polymer separators. Reproduced from Refs. [79,82,83] according to the copyright permissions or Creative Commons licenses of the original publications.

The representative SEM micrographs presented in Figure 10 illustrate the morphological characteristics of different ceramic-reinforced natural polymer separators discussed in this section. Although the separator architectures and ceramic nanofillers vary considerably, all studies aimed to improve electrolyte transport while maintaining adequate mechanical integrity and thermal stability. For a direct comparison of the best-performing formulations reported in each work, the key physicochemical, mechanical, and electrochemical properties are summarized in Table 9.

Collectively, these studies demonstrate that ceramic nanofillers play a key role in enhancing the performance of natural polymer separators, irrespective of the specific polymer matrix or fabrication strategy. Across cellulose fibers, cellulose acetate, and cellulose paper-based membranes, ceramic incorporation consistently improves electrolyte wettability, ionic conductivity, thermal stability, mechanical integrity, and electrochemical performance while maintaining efficient ion transport. However, the reported improvements arise from the combined effects of the natural polymer matrix, separator architecture, fabrication process, and ceramic composition. Consequently, rather than identifying a universally superior ceramic nanofiller, the available studies indicate that the performance of natural polymer–ceramic separators depends on the synergistic interaction between these design parameters.

Despite these promising results, it should be noted that most studies on multilayer ceramic-impregnated cellulose paper separators have been reported by the same research group. Further validation by independent studies, together with more comprehensive assessments of flexibility under repeated mechanical deformation, will be important to establish the broader applicability and reproducibility of these promising separator architectures.

## 4. Design Challenges and Future Opportunities

### 4.1. Broadening the Application Landscape

The studies reviewed in this work reveal a clear predominance of supercapacitor applications, particularly in the development of natural polymer–ceramic composite electrodes. In contrast, battery-related applications are more frequently represented in the electrolyte and separator sections, highlighting a comparatively limited exploration of natural polymer–ceramic composites as active electrode materials for flexible battery systems.

This imbalance identifies an important opportunity for future research. The structural versatility and processability of natural polymers, combined with the diverse functional properties offered by ceramic materials, could be further explored in flexible lithium-ion, sodium-ion, and zinc-based batteries, as well as metal–air batteries and hybrid battery–supercapacitor systems. Expanding the application landscape beyond the currently dominant supercapacitor configurations may help establish broader structure–property–performance relationships and clarify the potential of these composites across different energy-storage technologies.

The reviewed studies also show that carbon-based components are frequently incorporated alongside natural polymers and ceramic materials, particularly in electrode architectures, to enhance electrical conductivity and facilitate charge transport. Examples include carbon nanotube-, graphene-, and graphite-containing systems combined with ceramic electroactive phases. This broader class of multifunctional polymer–carbon–inorganic composites illustrates how the relative contribution and proportion of the different phases, together with processing conditions, can be used to tailor the electrical and structural properties of flexible materials. Similar strategies have also been explored in polymer–graphite composites for flexible electronic and electrochemical applications [97,98], providing an additional perspective for future materials design.

### 4.2. Moving from Bio-Based Materials to Demonstrated Sustainability

The use of natural polymers is frequently associated with sustainability; however, the presence of a bio-based component alone does not demonstrate the environmental sustainability of the resulting material or device. As highlighted in the reviewed literature, and also emphasized by Patel et al. [89], materials are often described as “green” without rigorous evaluation of aspects such as biodegradability, recyclability, precursor sourcing, toxicity, or end-of-life disposal.

The environmental advantages of natural polymer–ceramic energy-storage materials should therefore be assessed at the device level rather than inferred solely from the presence of a bio-based component. This is particularly important for composite systems that may incorporate synthetic polymers, carbon-based additives, metallic current collectors, complex electrolytes, or other components that influence the overall environmental profile of the final device.

Future studies should incorporate more comprehensive sustainability assessments, including life-cycle assessment, biodegradability and compostability studies, toxicity evaluation, recyclability, recovery and reuse of ceramic materials, and the potential reuse of complete devices or individual components. Quantifying the effective bio-based fraction of the final device would also provide a more realistic basis for evaluating its sustainability. Such analyses are particularly important given that, among the studies reviewed here, the actual environmental performance of the proposed materials was only explicitly assessed in one case, despite sustainability being frequently presented as a key motivation for their development.

### 4.3. Scalability, Reproducibility, and Industrial Feasibility

A further challenge concerns the transition from laboratory-scale demonstrations to practically relevant manufacturing. Many of the reported systems demonstrate promising electrochemical and mechanical properties at the proof-of-concept level; however, their large-scale production, processing reproducibility, material availability, and cost remain largely unexplored.

As also noted by Patel et al. [89], manufacturing scalability, cost considerations, and industrial feasibility are frequently overlooked in performance-centric studies. Future research should therefore move beyond proof-of-concept demonstrations and evaluate whether the proposed fabrication strategies can be translated to larger scales while maintaining consistent material properties and device performance.

Particular attention should be given to processing reproducibility, precursor availability, manufacturing cost, and compatibility with scalable fabrication approaches, including roll-to-roll or other continuous processing technologies where applicable. These considerations will be essential for determining whether high-performing natural polymer–ceramic composites can progress beyond laboratory demonstrations toward technologically and economically viable flexible energy-storage devices.

### 4.4. Expanding the Global Research Landscape

The bibliometric assessment further revealed a strong geographical concentration of research in Asia, particularly China, highlighting the leading role of this region in the development of natural polymer–ceramic nanocomposites for flexible energy storage. This concentration reflects the rapid development of the field but also suggests opportunities to broaden research activity and strengthen international collaboration.

Expanding research efforts across a wider range of geographical regions could promote access to different natural resources, locally available biopolymers and mineral precursors, and complementary processing technologies. Increased international collaboration may therefore contribute not only to a broader geographical distribution of research but also to greater material diversity, improved reproducibility across different laboratories, and the development of more globally applicable energy-storage technologies.

### 4.5. Opportunities for Artificial Intelligence and Machine Learning

The increasing diversity of natural polymer matrices, ceramic nanofillers, composite compositions, and device architectures also creates an opportunity for the application of artificial intelligence (AI) and machine-learning approaches. The large number of possible combinations makes conventional trial-and-error optimization increasingly time-consuming and resource-intensive.

AI- and machine-learning-assisted approaches could support the identification of relationships between composition, processing parameters, composite structure, mechanical properties, and electrochemical performance. In particular, these tools could help guide the selection of polymer–ceramic combinations and optimize parameters such as filler loading, component ratios, and processing conditions.

However, the usefulness of these approaches will depend strongly on the availability of sufficiently consistent and comparable datasets. The lack of standardized reporting and testing conditions identified throughout the present review therefore represents not only a challenge for direct comparison between studies but also a limitation for future data-driven materials design. Greater standardization in the reporting of composition, fabrication parameters, electrochemical performance, and mechanical behaviour would consequently facilitate both conventional comparative analyses and the future implementation of AI-assisted materials discovery and optimization.

## 5. Conclusions

Natural polymer–ceramic nanocomposites represent a promising materials platform for the development of flexible and wearable energy-storage devices by combining the structural versatility and flexibility of natural polymers with the electrochemical, ionic, thermal, and mechanical functionalities provided by ceramic nanofillers. This review shows that cellulose-based materials and chitosan are the most extensively investigated natural polymer matrices, while metal oxides and other functional ceramic materials have been incorporated into a broad range of architectures for electrodes, electrolytes, and separators. Among these components, electrode materials remain the most widely investigated, with flexible supercapacitors representing the dominant device application.

Overall, the future development of natural polymer–ceramic nanocomposites for flexible energy storage will require progress beyond the optimization of individual electrochemical performance metrics. Expanding their application to a broader range of energy-storage technologies, rigorously assessing their environmental sustainability, and addressing scalability, reproducibility, and data-driven materials design are important steps toward practical implementation. A more integrated evaluation of electrochemical performance, mechanical durability, environmental impact, and manufacturability may ultimately facilitate the transition of these materials from laboratory-scale demonstrations to viable flexible and wearable energy-storage technologies.

## Figures and Tables

**Table 1:** Overview of the natural polymer–ceramic nanocomposite systems discussed in this review, including the natural polymer matrix, additional polymer(s), ceramic nanofiller, device component, and target energy storage application.

Ref.	Natural Polymer	Additional Polymer(s)	Ceramic Nanofiller	Device Component	Energy Storage Application
[44]	Rice paper (cellulose)	-----	MnO_2_	Electrode	SC
[45]	Cellulose nanocrystals	-----	MnO_2_	Electrode	SC
[46]	Alginate	PEDOT	MnO_2_	Electrode	SC
[47]	Chitosan	-----	MnO_2_	Electrode	SC
[48]	Cellulose nanofibers	-----	MnO_2_	Electrode	SC
[49]	TEMPO-oxidized cellulose nanofibres	PANI	MnO_2_	Electrode	SC
[50]	Cellulose nanocrystals	-----	MnO_2_	Electrode	SC
[51]	Cellulose nanofibers	-----	Fe_3_O_4_	Electrode	SC
[52]	Bacterial cellulose	Polypyrrole	Fe_3_O_4_	Electrode	SC
[53]	Cellulose (recovered from diaper waste)	-----	PbFe_11_CrO_19_	Electrode	SC
[54]	Cellulose nanofibers	-----	Fe_3_O_4_	Electrode	SC
[55]	Cellulose nanofibers	-----	Fe_3_O_4_ and MnFe_2_O_4_	Electrode	SC
[56]	Cellulose nanofibers	-----	Fe_3_O_4_	Electrode	SC
[57]	Bacterial nanocellulose	PEDOT and PSS	SnO_2_	Electrode	SC
[58]	Cellulose nanofibers	-----	SnO_2_	Electrode	SC
[59]	Microfibrillated cellulose	-----	SnO_2_	Electrode	SC
[60]	Microfibrillated cellulose	-----	SnO_2_	Electrode	SC
[61]	Cellulose nanofibers	-----	Co_3_O_4_	Electrode	SC
[62]	Cellulose nanofibers	-----	Co_3_O_4_	Electrode	SC
[63]	Lignocellulosic fibers	-----	ZnO/MnO_2_ and ZnO/TiO_2_	Electrode	SC
[64]	Cellulose nanofibers	-----	ZnO	Electrode	SC
[65]	Cellulose nanofibers	-----	ZnO/boron nitride nanotubes	Electrode	SC
[66]	Cellulose	-----	TiO_2_	Electrode	SIBs
[67]	Cellulose acetate	-----	TiO_2_	Electrode	SC
[68]	Bacterial cellulose	PPy	CuO	Electrode	SC
[69]	Chitosan	PVDF	CuO	Electrode	SC
[70]	Coconut fiber	PMMA and pectin	MgTiO_3_	Electrode	SC
[71]	Chitosan	-----	MoS_2_	Electrode	SC
[72]	Chitosan	PVDF	Ni-doped CeO_2_	Electrode	SC
[73]	Cellulose acetate and chitosan	PANI	NiO and Fe_3_O_4_	Electrode	SC
[74]	Cellulose	Epoxy resin (E44)	Halloysite nanotubes (HNTs)	Electrolyte (ionogel)	SC
[75]	Cellulose	PVDF and PEGDA	Li_1.5_Al_0.5_Ge_1.5_(PO_4_)_3_ (LAGP)	Electrolyte (membrane)	LIBs
[76]	Cellulose	ETPTA	Li_6.4_La_3_Zr_1.4_Ta_0.6_O_12_ (LLZTO)	Electrolyte (membrane)	LIBs
[77]	Chitosan	-----	SiO_2_	Electrolyte (ionogel)	SC
[78]	Carboxymethyl cellulose	PANa	SiO_2_	Electrolyte	ZABs
[79]	Cellulose fibers	-----	HAp	Separator	LIBs
[80]	Cellulose acetate	PVA	TiO_2_	Separator	ZABs
[81]	Cellulose paper	Chitosan + PVA	SiO_2_	Separator	LIBs
[82]	Cellulose paper	PVDF and SBR	BaTiO_3_	Separator	LIBs + SC
[83]	Cellulose paper	PVDF and SBR	BaTiO_3_	Separator	SIBs
[84]	Cellulose paper	PVDF and SBR	Al_2_O_3_, BaTiO_3_ and ZrO_2_	Separator	LIBs

**Table 2:** Comparison of the key electrochemical properties and flexibility-related mechanical performance of selected MnO_2_-based natural polymer electrode formulations discussed in this review. For studies investigating multiple formulations, only the formulation identified by the authors as the best-performing is included.

Ref.	Selected Formulation	Capacitance	Energy Density (Wh kg^−1^)	Power Density (kW kg^−1^)	Cycling Stability	[Mechanical]/Flexibility Performance
[44]	SWCNT/MnO_2_/Paper §	C = 260.2 F g^−1^	9.0	59.7	88% (3000 cycles)	Negligible loss after >100 bending cycles
[45]	CNC/MnO_2_ §	C_CS_ = 2.14 mF cm^−2^	Not reported	Not reported	97.81% (1000 cycles)	83% shape recovery after 400 compression cycles; stable CV response up to 80% compressive strain
[46]	PEDOT/Alg/CNPs/MnO_2_ (30 min MnO_2_ deposition) †	C_CS_ = 42 mF cm^−2^	Not reported	Not reported	≈80% (3000 cycles)	Electrochemical activity largely recovered after complete folding; similar energy density before and after bending
[47]	Chitosan/GO/MnO_2_ (20 h MnO_2_ deposition) §	C_CS_ = 1050 mF cm^−2^	Not reported	Not reported	≈74.1% (10,000 cycles)	Stable electrochemical response under repeated finger pressing and bending/folding
[48]	CNFs/CMTs/MnO_2_ (30 min MnO_2_ deposition) §	C_CS_ = 934 mF/cm^2^	43.10 Wh/kg	166.67 W/kg	≈85% (10,000 cycles)	Stable CV response at 90° and 180° bending; 93.6% capacitance retention after 500 folding cycles
[49]	TOCNs/CNTs/PANI/MnO_2_ §	C = 1108 mF/cm^2^	Not reported	Not reported	82.0% (5000 cycles)	95% retention after 200 bending cycles; 90% after 10 cut/healing cycles
[50]	MnO_2_/CNCs/graphite (Deposition at 150 °C) §	C_CS_ = 149 mF/cm^2^	Not reported	Not reported	85.27% (15,000 cycles)	Not reported

† three-electrode measurement and § complete device.

**Table 3:** Comparison of the key electrochemical properties and flexibility-related mechanical performance of selected iron-based natural polymer electrode formulations discussed in this review. For studies investigating multiple formulations, only the formulation identified by the authors as the best-performing is included.

Ref.	Selected Formulation	Capacitance	Energy Density (Wh kg^−1^)	Power Density (kW kg^−1^)	Cycling Stability	[Mechanical]/Flexibility Performance
[51]	CNF/MWCNT/RGO/Fe_3_O_4_ ([Fe^3+^] = 0.08 mol L^−1^) §	C_CS_ = 5820 mF/cm^3^	Not reported	Not reported	94.7% (5000 cycles)	Stable CV profiles under different bending angles
[52]	BC/Fe_3_O_4_/PPy ([PPy] = 0.2 mol L^−1^) †	C_CS_ = 5430 mF/cm^2^	Not reported	Not reported	81.4% (3500 cycles)	[Tensile strength of 11 MPa]
[53]	Cellulose/graphene/PbFe_11_CrO_19_ §	C = 1894.8 F g^−1^	164.4	Not reported	90.3% (2000 cycles)	88.2% capacitance retention at a bending radius of 2.8 cm
[54]	Fe_3_O_4_/MWCNT/CNF (MWCNT@Fe_3_O_4_/MWCNT/CNF—7:1:2 mass ratio) §	C = 107.0 F g^−1^ C_CS_ = 2940 mF cm^−2^	38.0	0.405	>90% (5000 cycles)	98% capacitance retention after five bending cycles at 180°
[55]	Fe_3_O_4_/MWCNT/CNF//MnFe_2_O_4_/MWCNT/CNF (MWCNT@Fe_3_O_4_/MWCNT/CNF and MWCNT@MnFe_2_O_4_/MWCNT/CNF, 7:1:2 mass ratio; 250 μL of each electrode ink) §	C_CS_ = 309.8 mF cm^−2^	Not reported	Not reported	>95% (5000 cycles)	96.3% capacitance retention at 180° bending
[56]	Fe_3_O_4_/CNF (Fe_3_O_4_:CNF = 1:1, 100 °C) §	C = 188.3 F g^−1^	33.9	0.54	94% (8000 cycles)	[62 MPa tensile strength; 79.2% elongation at break;] stable electrochemical response at 180° bending

† three-electrode measurement and § complete device.

**Table 4:** Comparison of the key electrochemical properties and flexibility-related mechanical performance of selected SnO_2_-based natural polymer electrode formulations discussed in this review. For studies investigating multiple formulations, only the formulation identified by the authors as the best-performing is included.

Ref.	Selected Formulation	Capacitance	Energy Density (Wh kg^−1^)	Power Density (kW kg^−1^)	Cycling Stability	[Mechanical]/Flexibility Performance
[57]	PEDOT:PSS/SnO_2_/rGO/BNC §	C = 603 F g^−1^	Not reported	Not reported	89.8% (2500 cycles)	Stable CV profiles under different bending angles
[58]	CNF/RGO/SnO_2_ (F-CRA) †	C_CS_ = 4300 mF cm^−2^	Not reported	Not reported	60.47% (2000 cycles)	No mechanical or bending characterization reported; electrode flexibility not experimentally demonstrated
[59]	rGO/SnO_2_/MFC (rGO/SnO_2_:MFC = 66:34 wt%) §	C = 53 F g^−1^	Not reported	Not reported	>90% (1000 cycles)	[Young’s modulus of 213 MPa and tensile strength of 0.9 MPa;] >80% capacitance retained at high bending angles
[60]	MFC/SnO_2_ (12 wt% SnO_2_) †	C = 486.38 F g^−1^	Not reported	Not reported	95% (40 cycles)	Flexible and foldable self-standing film visually demonstrated; no quantitative mechanical characterization or electrochemical bending tests reported

† three-electrode measurement § complete device.

**Table 5:** Comparison of the key electrochemical properties and flexibility-related mechanical performance of selected Co_3_O_4_-based natural polymer electrode formulations discussed in this review. For studies investigating multiple formulations, only the formulation identified by the authors as the best-performing is included.

Ref.	Selected Formulation	Capacitance	Energy Density (Wh kg^−1^)	Power Density (kW kg^−1^)	Cycling Stability	[Mechanical]/Flexibility Performance
[61]	Co_3_O_4_/CNF (Co_3_O_4_:CNF = 1:1) §	C = 80 F g^−1^	10	0.9	Not reported for the flexible solid-state device; ~94% (5000 cycles) in the aqueous KOH electrolyte	Stable CV profiles under bending at different angles and after 50 bending cycles
[62]	Co_3_O_4_/CNF (60 wt% PCP-400) §	C = 52.74 F g^−1^	18.75	8.00	64% (2000 cycles)	Free-standing film visually demonstrated to withstand bending and rolling; no quantitative mechanical characterization or electrochemical testing under bending reported

§ complete device.

**Table 6:** Comparison of the key electrochemical properties and flexibility-related mechanical performance of selected ZnO- and TiO_2_-based natural polymer electrode formulations discussed in this review. For studies investigating multiple formulations, only the formulation identified by the authors as the best-performing is included.

Ref.	Selected Formulation	Capacitance	Energy Density (Wh kg^−1^)	Power Density (kW kg^−1^)	Cycling Stability	[Mechanical]/Flexibility Performance
[63]	ZnO/MnO_2_/LC and ZnO/TiO_2_/LC †	Not reported	Not reported	Not reported	ZnO/MnO_2_/LC: ~70% ZnO/TiO_2_/LC: ~87% (100 cycles)	No quantitative mechanical characterization or electrochemical evaluation under mechanical deformation reported
[64]	CNF/ZnO (68 wt% ZnO) §	C = 140 F g^−1^	25.2	1.08	92% (8000 cycles)	No significant decay in CV current response or integrated area after 100 bending cycles at 90°
[65]	BNTT/CNF/ZnO §	C = 94 F g^−1^	30.7	1.35	97% (5000 cycles)	CV response remained nearly unchanged under bending angles from 0° to 180
[66]	TiO_2_/CNT/cellulose (75 wt% TiO_2_) ‡	N/A ---- Specific capacity: 227 mAh g^−1^ at 0.15 mA cm^−2^; 72 mAh g^−1^ at 10 mA cm^−2^	N/A	N/A	81.5% (9000 cycles)	[Tensile strength: 46 ± 2.4 MPa; survived >3000 folding cycles at a bending radius of 0.6 mm]
[67]	TiO_2_/CA (1.0 wt% TiO_2_) *	C = 98.15 F g^−1^	23.03	1.04	88% (5000 cycles)	[Tensile strength: 39.2 MPa; Young’s modulus: 8016 MPa; elongation at break: 0.299%;] no electrochemical evaluation under mechanical deformation reported

† three-electrode measurement, ‡ half-cell configuration, * two-electrode configuration, and § complete device.

**Table 7:** Comparison of the key electrochemical properties and flexibility-related mechanical performance of other ceramic-based natural polymer electrode formulations discussed in this review. For studies investigating multiple formulations, only the formulation identified by the authors as the best-performing is included.

Ref.	Selected Formulation	Capacitance	Energy Density (Wh kg^−1^)	Power Density (kW kg^−1^)	Cycling Stability	Mechanical/Flexibility Performance
[68]	PPy/CuO/BC (1 wt% Cu(Ac)_2_) *	C = 601 F g^−1^	48.2	0.0858	64% (300 cycles)	Bent to 180° and repeatedly folded without fracture; no quantitative mechanical characterization or electrochemical evaluation under deformation reported
[69]	Chitosan/CuO (1:1 CS:CuO, wt/wt) †	C = 325 F g^−1^	11.28	0.92957	>99% (10,000 cycles)	No mechanical or flexibility-related characterization reported; electrochemical testing performed using a slurry-coated Ni foam electrode
[70]	Coconut fiber/graphene/ MgTiO_3_ §	C = 807 F g^−1^	161.4	0.0162	62–68% (1000 cycles)	91% capacitance retention after 500 bending cycles
[71]	Chitosan/MoS_2_ (1.5 wt% MoS_2_) *	C = 345.8 F g^−1^	22.2	0.544	96% (5000 cycles)	[Tensile strength: 43.3 MPa; Young’s modulus: 11,162 MPa; elongation at break: 1.05%]
[72]	Chitosan/Ni-CeO_2_ §	C = 190 F g^−1^	65	0.8	90% (5000 cycles)	94% capacitance retention under 180° bending
[73]	Chitosan/CA/PANI/rGO/NiO/Fe_3_O_4_ (15 wt% rGO/NiO/Fe_3_O_4_) §	C_CS_ = 5.5 mF cm^−2^	Not reported	Not reported	71.26% (1000 cycles)	Only slight changes in the CV response under 90° and 120° bending; no quantitative retention or repeated bending-cycle test reported

† three-electrode measurement, * two-electrode configuration, and § complete device.

**Table 8:** Comparison of the key physicochemical, mechanical, and electrochemical characteristics of the ceramic-reinforced natural polymer electrolytes discussed in this review. For studies investigating multiple formulations, only the electrolyte identified by the authors as the optimal or best-performing formulation is presented.

Ref.	Selected Formulation	Ionic Conductivity (mS cm^−1^)	Tensile Strength (MPa)	Thermal Stability (°C)	Electrochemical Stability Window (V)	Capacity Retention (%)
[74]	Cellulose (5 wt%)/HNTs (15 wt%)/E44 (2.5 wt%)/CAN (0.25 wt%)	1.6	3.2	200	Not reported	94.3 (5000 cycles)
[75]	Cellulose mesh/LAGP/PVDF + PEGDA/FEC/LiTFSI	0.112	4.68	≈250 °C	4.7	Not reported
[76]	Cellulose mesh/poly(ETPTA)/LLZTO	0.652	Not reported	Not reported	4.82	91.6 (200 cycles)
[77]	Chitosan (8 wt%)/nano-SiO_2_ (2 wt%)	24.3	Not reported	Not reported	1.0	Not reported
[78]	Carboxymethyl celulose/PANa/SiO_2_	276.51	3.62	Not reported	Not reported	Not reported

**Table 9:** Comparison of the key physicochemical, mechanical, and electrochemical characteristics of the ceramic-reinforced natural polymer separators discussed in this review. For studies investigating multiple formulations, only the separator identified by the authors as the optimal or best-performing formulation is presented.

Ref.	Selected Formulation	Electrolyte Uptake (%)	Porosity (%)	Ionic Conductivity (mS cm^−1^)	Tensile Strength (MPa)	Thermal Stability (°C)	Capacity Retention (%)
[79]	CFs/HAp	253	81 (Gurley: 83 s)	3.07	13.21	200 (no shrinkage)	92.3 (100 cycles)
[80]	CA/PVA/2%TiO_2_ +5 wt% of GA	285.3	68.2	10.36	9.6	Not accessed	Not accessed
[81]	Cellulose paper/Chitosan/PVA/1.0 wt% SiO_2_	147	52.62–55.18 (Gurley: 21.95 s)	0.58	34.86	>200 (no shrinkage)	Not reported
[82]	Cellulose paper/PVDF/(SBR/BaTiO_3_) (2 wt/v% SBR lamination)	270.21	Not reported (Gurley: 120 s)	0.19	28.21 (TD) 50.15 (MD)	200 (no shrinkage)	Not reported
[83]	Cellulose paper/PVDF/(SBR/BaTiO_3_) (0.75 wt/v% SBR lamination)	152–158	Not reported (Gurley: 13.0 s)	0.27	27.57 (TD) 48.78 (MD)	200 (no shrinkage)	62 (240 cycles)
[84]	Cellulose paper/PVDF/(SBR/2.0 w/v%ZrO_2_)	287.17	Not reported (Gurley: 13.16 s)	1.08	27.88 (TD) 40.08 (MD)	>200 (no shrinkage)	53.89 (300 cycles)

Abbreviations: TD, transverse direction; MD, machine direction.

## Data Availability

No new data were created in this study. Data sharing does not apply to this article.

## Abbreviations

The following abbreviations are used in this manuscript:

AB	acetylene black
AI	artificial intelligence
BNC	bacterial nanocellulose
CA	cellulose acetate
CF	cellulose fibre
CNF	cellulose nanofibre
CNC	cellulose nanocrystal
CMC	carboxymethyl cellulose
CNP	carbon nanoparticle
CNT	carbon nanotube
CV	cyclic voltammetry
EDLC	electric double-layer capacitor
FESD	flexible energy-storage device
GCD	galvanostatic charge–discharge
GO	graphene oxide
HAp	hydroxyapatite
HNT	halloysite nanotube
LAGP	Li_1.5_Al_0.5_Ge_1.5_(PO_4_)_3_
LIB	lithium-ion battery
LLZTO	Li_6.4_La_3_Zr_1.4_Ta_0.6_O_12_
MFC	microfibrillated cellulose
MD	machine direction
MWCNT	multiwalled carbon nanotube
NFC	nanofibrillated cellulose
PANI	polyaniline
PANa	sodium polyacrylate
PCP	porous Co_3_O_4_ polyhedron
PDMS	polydimethylsiloxane
PEDOT	poly(3,4-ethylenedioxythiophene)
PE	polyethylene
PEGDA	poly(ethylene glycol) diacrylate
PEO	polyethylene oxide
PP	polypropylene
PPy	polypyrrole
PSS	poly(styrene sulfonate)
PVA	poly(vinyl alcohol)
PVDF	poly(vinylidene fluoride)
rGO	reduced graphene oxide
SC	supercapacitor
SBR	styrene–butadiene rubber
SIB	sodium-ion battery
SWCNT	single-walled carbon nanotube
TD	transverse direction
TOCN	TEMPO-oxidized cellulose nanofibre
ZAB	zinc–air battery
